# School Absenteeism and Self-Harm or Suicidal Outcomes Among Children, Adolescents, and School-Aged Young People: A Systematic Review and Meta-Analysis

**DOI:** 10.3390/children13101373

**Published:** 2026-10-10

**Authors:** Lazzat Zhamaliyeva, Zhadyra Zhalgasbaeva

**Affiliations:** 1 Department of General Practice No. 2, West Kazakhstan Marat Ospanov Medical University, Aktobe 030000, Kazakhstan; lzhamalieva@gmail.com; 2 Department of Children’s Diseases No. 2, West Kazakhstan Marat Ospanov Medical University, Aktobe 030000, Kazakhstan

**Keywords:** school absenteeism, truancy, school refusal, self-harm, suicidal ideation, suicide plan, suicide attempt, adolescents, children, meta-analysis

## Abstract

**Highlights:**

**What are the main findings?**

- School attendance-related problems were associated with higher odds of suicidal ideation, suicide plan, and suicide attempts among children, adolescents, and school-aged young people.
- The quantitative evidence primarily reflected truancy, skipped school, and unauthorised absence, while evidence for chronic absence, school refusal, and absence because of feeling unsafe was more limited.

**What are the implications of the main findings?**

- School absenteeism should be interpreted as a potential marker of psychosocial vulnerability rather than as evidence of a direct causal relationship or a standalone suicide risk prediction tool.
- Attendance concerns should prompt supportive, non-punitive assessment that considers mental health symptoms, bullying, family adversity, school connectedness, and safety concerns.

**Abstract:**

**Background**: School absenteeism may indicate psychosocial vulnerability among children and adolescents. Although absenteeism, truancy, poor attendance, and school avoidance have been linked with self-harm and suicidal behaviours, the strength and certainty of this association require systematic synthesis. **Objective**: This systematic review and meta-analysis evaluated the association between school absenteeism and self-harm or suicidal outcomes among children, adolescents, and school-aged young people. **Methods**: The review was reported using PRISMA 2020 and MOOSE guidance and was registered in PROSPERO before journal submission (CRD420261472681). PubMed/MEDLINE, Web of Science Core Collection, and Scopus were searched for updated records from 7 June 2018 to 27 May 2026. Studies from a previous systematic review were reassessed against the current eligibility criteria. Eligible studies were quantitative observational studies of school-aged populations that measured school absenteeism or related attendance problems and reported self-harm, suicidal ideation, suicide plan, suicide attempt, suicidal behaviour, or suicide death. Risk of bias was evaluated using an adapted observational exposure-study framework, and certainty of evidence was assessed using an adapted GRADE approach. **Results**: Forty-seven studies were included: 15 newly identified studies and 32 studies retained from the previous review. The evidence was geographically diverse but was dominated by cross-sectional school-based surveys. Random-effects meta-analyses showed that attendance-related problems, particularly truancy, skipped school, or unauthorised absence, were associated with higher odds of suicidal ideation (12 estimates; OR = 1.32, 95% CI: 1.19–1.46; I^2^ = 63.3%), suicide plan (6 estimates; OR = 1.31, 95% CI: 1.21–1.42; I^2^ = 0.0%), and suicide attempt (14 estimates; OR = 1.60, 95% CI: 1.41–1.81; I^2^ = 69.4%). Risk of bias was moderate in 26 studies and serious in 21 studies; no study was rated as having low overall risk of bias. **Conclusions**: Low-certainty observational evidence suggests that attendance-related problems, particularly truancy, skipped school, or unauthorised absence, are associated with increased odds of suicidal ideation, suicide plan, and suicide attempt. Absenteeism should be interpreted as a potential marker of psychosocial vulnerability rather than as a standalone suicide risk prediction tool or evidence of causality. Evidence for chronic or persistent absence, school refusal, and school avoidance remains more limited.

## 1. Introduction

Self-harm and suicidal behaviours among children and adolescents are major public health concerns. Globally, suicide is the third leading cause of death among people aged 15–29 years, and many more young people experience suicidal ideation, suicide planning, suicide attempts, or non-fatal self-harm [1]. Adolescence is a particularly vulnerable developmental period, during which biological, psychological, social, family, and school-related stressors may interact to increase the risk of emotional distress and suicidal behaviour. Mental health problems are common in this age group; globally, approximately one in seven 10–19-year-olds experiences a mental disorder, and depression, anxiety, and behavioural disorders are among the leading contributors to adolescent morbidity [2].

Schools are central social, developmental, and educational environments for children and adolescents. Beyond academic learning, regular school participation provides structure, peer relationships, adult supervision, access to pastoral and safeguarding systems, and opportunities for early identification of mental health problems [3,4]. Conversely, school difficulties such as poor school connectedness, bullying, violence exposure, academic disengagement, disciplinary problems, and truancy have been repeatedly associated with adolescent self-harm and suicidal outcomes [3,5,6,7,8,9,10,11,12,13,14,15]. These findings suggest that school-related indicators may be important markers of psychosocial vulnerability and may help identify young people who require support.

School absenteeism is one such indicator. It is a broad construct that may include truancy, unauthorised absence, missed classes, skipped school, poor attendance, chronic or persistent absence, school refusal, school avoidance, and absence because of feeling unsafe at school. These forms of non-attendance may have different meanings. Truancy may reflect disengagement, rule-breaking, weak supervision, peer influence, or broader behavioural risk, whereas school refusal or avoidance may be more closely linked to anxiety, depression, bullying, trauma, or perceived threat. Persistent absence recorded in administrative data may also reflect illness, family adversity, exclusionary processes, socioeconomic disadvantage, neurodevelopmental needs, or unmet educational support. The current review defines school absenteeism broadly to include truancy, unauthorised absence, missed classes, skipped school, poor attendance, chronic or persistent absence, absence because of feeling unsafe, and school refusal or avoidance when operationalised as non-attendance [4]. For this review, school absenteeism was therefore treated as an umbrella term covering several attendance-related problems. The pooled analyses mainly reflect evidence for truancy, skipped school, and unauthorised absence, because these were the exposure definitions most often available for quantitative synthesis. Findings for chronic or persistent absence, school refusal or avoidance, and absence related to feeling unsafe at school were more limited and were considered separately where possible.

Several mechanisms may plausibly link absenteeism with self-harm and suicidal outcomes. First, absenteeism may be an early marker of underlying emotional or behavioural problems, including depression, anxiety, substance use, trauma-related symptoms, neurodevelopmental difficulties, or previous self-harm [3,5,6,7,8,9,10,11,12,13,14,15]. Second, repeated absence may reduce exposure to protective school factors, including supportive peer relationships, trusted adults, routine, supervision, and opportunities for help-seeking [3,4]. Third, absenteeism may reflect adverse school environments, including bullying, peer victimisation, violence, social exclusion, or feeling unsafe at school [3,4,14,16]. Fourth, absence may contribute to academic difficulties, conflict with parents or teachers, disciplinary involvement, and feelings of failure or hopelessness. Finally, the association may be bidirectional: suicidal ideation, self-harm, or suicide attempts may lead to absence, while absence may further increase isolation and psychosocial vulnerability.

Although many primary studies have examined associations between school absenteeism and suicidal or self-harm outcomes, the evidence base remains heterogeneous. Studies differ in their definitions of absenteeism, reference periods, measurement sources, outcome definitions, study designs, geographical settings, and adjustment for confounding. Much of the literature consists of cross-sectional school-based surveys, but a smaller number of longitudinal, case–control, and record-linkage studies suggest that attendance problems may precede later self-harm, suicide attempts, or suicide death [7,17,18,19,20,21,22]. This distinction is important because cross-sectional designs cannot establish temporal order and are vulnerable to reverse causation, whereas longitudinal and administrative studies may provide stronger evidence about whether absenteeism functions as an early warning indicator.

A synthesis focused specifically on absenteeism is therefore needed. Previous research has often considered truancy or poor attendance as one variable within broader models of adolescent risk, alongside bullying, substance use, family adversity, school connectedness, and mental health symptoms [4]. However, absenteeism is routinely recorded in many education systems and may be actionable in school, clinical, and public health settings. If absenteeism is consistently associated with self-harm or suicidal outcomes, attendance data may contribute to earlier identification of young people experiencing psychosocial distress. At the same time, it is important to determine whether this association is robust across outcome types, exposure definitions, study designs, and levels of confounder adjustment.

This review aimed to summarise quantitative evidence on whether school absenteeism and related attendance problems are associated with self-harm or suicidal outcomes in children, adolescents, and school-aged young people. The outcomes of interest were suicidal ideation, suicide plan, suicide attempt, suicidal behaviour, self-harm, and suicide death. When sufficient comparable data were available, outcome-specific meta-analyses were performed for suicidal ideation, suicide plan, and suicide attempt. We also examined study characteristics, risk of bias, heterogeneity, subgroup patterns, and sensitivity analyses to support interpretation of the evidence.

## 2. Methods

### 2.1. Protocol and Registration

This review was planned and reported using the PRISMA 2020 framework and the MOOSE recommendations for meta-analyses of observational studies. The review protocol was registered in PROSPERO before journal submission (CRD420261472681; registered 7 August 2026). Registration was completed after initiation of the review and after the updated database search had been conducted; therefore, the review is not described as prospectively registered. As this study used data from published reports and de-identified secondary sources, separate ethics approval was not required. The completed PRISMA 2020 checklist is provided as Supplementary File S1.

The review question was structured using a PECOS framework. The population comprised children, adolescents, and school-aged young people. The exposure was school absenteeism or closely related attendance problems. Comparators were students with no absence, lower levels of absence, or the reference group defined in each primary study. Outcomes were self-harm, suicidal ideation, suicide plan, suicide attempt, suicidal behaviour, and suicide death. Eligible study designs were quantitative observational designs, including cross-sectional, cohort, case–control, nested case–control, record-linkage, and secondary analyses of population survey data.

### 2.2. Eligibility Criteria

A study was considered eligible when it satisfied the prespecified population, exposure, outcome, and study-design criteria: (1) reported quantitative data; (2) included children, adolescents, or school-aged young people, or reported data for a relevant school- or college-attending youth subgroup; (3) measured school absenteeism or a closely related attendance exposure; (4) reported self-harm, suicidal ideation, suicide plan, suicide attempt, suicidal behaviour, or suicide death as an outcome; and (5) provided an effect estimate, such as an odds ratio, risk ratio, hazard ratio, prevalence ratio, or sufficient numerical data to calculate an effect estimate.

School absenteeism was defined broadly to include truancy, unauthorised absence, missed classes, skipped school, poor attendance, chronic or persistent absence, absence from school because of feeling unsafe, and school refusal or school avoidance when these were operationalised as non-attendance. Studies that measured school exclusion, suspension, expulsion, school dropout, or broader school disengagement were eligible only when an absenteeism-specific exposure or estimate was reported separately; studies in which these constructs were the sole attendance-related exposure were excluded.

Because attendance-related constructs may reflect different mechanisms, exposure definitions were classified for reporting and subgroup and sensitivity analyses into: (1) truancy, skipped school, or unauthorised absence; (2) chronic, persistent, or administratively recorded poor attendance; (3) absence because of feeling unsafe at school; (4) school refusal or school avoidance operationalised as non-attendance; and (5) other attendance-related measures. Eligible outcomes included non-suicidal self-injury, deliberate self-harm, self-poisoning or self-injury presentations, suicidal ideation, suicide planning, suicide attempts, suicidal behaviour composite measures, and suicide death. Outcomes could be self-reported, clinically assessed, recorded in administrative health data, or identified from mortality records. Studies in which self-harm or suicidal outcomes were measured only in adulthood were excluded unless the adult follow-up was part of a longitudinal design in which school absenteeism was measured during childhood or adolescence.

Reviews, editorials, commentaries, conference abstracts without sufficient data, case reports, case series without a comparison group or association estimate, qualitative studies, and intervention studies without relevant baseline association data were excluded. Studies conducted exclusively in adult populations, or in highly selected clinical or inpatient samples with no extractable school-aged attendance association, were also excluded. Studies including participants older than 18 years were retained only when the population consisted of school-, college-, or education-attending young people and when the exposure was measured in relation to school or education attendance. Where data permitted, sensitivity analyses were planned to examine the influence of studies including participants older than 18 years. No restrictions were applied by country or setting. Publication-year coverage was achieved using a two-stage approach: studies published before 7 June 2018 were obtained from the previous systematic review and re-screened against the current eligibility criteria, while updated database searches covered records published from 7 June 2018 to 27 May 2026. Where multiple publications used the same or overlapping dataset, the most complete report was retained for each outcome and exposure unless publications reported distinct outcomes, non-overlapping samples, different follow-up periods, or disaggregated estimates required for the review. In cases of overlap, priority was given to the study with the largest relevant sample, the clearest absenteeism exposure definition, and the most appropriate adjustment for confounding.

### 2.3. Role of the Previous Systematic Review

Studies published before the updated search period were identified from the previous systematic review by Epstein et al. [4]. The previous review reported searches of MEDLINE, PsycINFO, Embase, ERIC, and the British Education Index up to 6 June 2018. For the present review, studies from the previous review were re-screened against the current eligibility criteria. Thirty-two studies met the current inclusion criteria and were retained. Inclusion in the previous review was not treated as automatic eligibility. Each study was reassessed using the current PECOS criteria. Overlap between the previous review and updated database searches was checked by author, year, title, country, dataset, exposure, and outcome. We relied on the previous review for studies published before 7 June 2018 and did not re-run electronic searches for the earlier period; this reliance is acknowledged as a limitation.

### 2.4. Information Sources and Search Strategy

An updated electronic literature search was conducted in PubMed/MEDLINE, Web of Science Core Collection, and Scopus from 7 June 2018 to 27 May 2026. The updated search was limited to these three databases and was designed to identify studies published after the previous review. The omission of additional discipline-specific databases, including PsycINFO, Embase, ERIC, CINAHL, and Education Source, was reconsidered during revision. No additional database searches were added at this stage because the present review was designed as an update of the previous review, which had searched MEDLINE, PsycINFO, Embase, ERIC, and the British Education Index up to 6 June 2018, and because the updated search used three broad biomedical and multidisciplinary databases. This limitation is acknowledged explicitly and was considered when interpreting the completeness and certainty of the evidence base.

Reference lists of included papers and relevant reviews were also checked for additional eligible studies. When necessary, article records were cross-checked against journal websites and database records to confirm publication details, population characteristics, and availability of extractable effect estimates. Search results were exported to EndNote, and duplicate records were identified and removed before title-and-abstract screening.

The search strategy combined terms for school attendance, self-harm or suicidality, and children or adolescents. Attendance-related terms included absenteeism, absence, attendance, truancy, truant, skipped school, missed school, missed classes, school refusal, school avoidance, chronic absence, persistent absence, and poor attendance. Outcome-related terms included self-harm, self-injury, non-suicidal self-injury, NSSI, deliberate self-harm, self-poisoning, suicidality, suicidal ideation, suicide plan, suicide attempt, suicidal behaviour, and suicide death. Population terms included child, adolescent, youth, student, pupil, school, secondary school, high school, and college.

Search terms were adapted for the syntax and controlled vocabulary of each database. A representative search structure was: (school OR student OR pupil OR adolescent OR child OR youth) AND (absenteeism OR absence OR attendance OR truancy OR truant OR skipped school OR missed class* OR school refusal OR chronic absence OR persistent absence) AND (suicid* OR self-harm OR self-injur* OR NSSI OR suicide attempt* OR suicidal ideation OR suicide plan*). The full database-specific search strategies, including execution dates, date limits, and filters used, are reported in Supplementary Table S1.

No methodological design filters were applied at the search stage unless required by the database interface. Search results were not restricted by geographical region. Full-text eligibility was limited to studies available in English.

### 2.5. Screening and Eligibility Assessment

Following deduplication, records retrieved from the updated database search were screened by title and abstract using the predefined eligibility criteria.

Records were excluded at this stage if they clearly did not include a relevant school attendance-related exposure, a relevant self-harm or suicidal outcome, a child or adolescent population, or quantitative data suitable for estimating an exposure–outcome association. Reports judged potentially eligible, or for which eligibility was unclear from the title and abstract, were retrieved for full-text assessment.

The same eligibility criteria were then applied during full-text assessment. Reasons for exclusion at the full-text stage were recorded and grouped into prespecified categories, including wrong exposure, wrong population, wrong outcome, wrong study design, duplicate or overlapping data, and no extractable absenteeism-specific effect estimate. Studies included in the previous systematic review were also re-assessed against the eligibility criteria of the present review and retained when they met the current scope.

Study selection decisions were checked by a second reviewer. Disagreements were resolved through discussion and, where necessary, by consultation with a third reviewer. The study selection process was documented using a PRISMA flow diagram.

### 2.6. Data Extraction

A structured extraction form developed for this review was used to collect study-level, exposure, outcome, and effect-estimate data. Extracted study characteristics included first author, publication year, country, data source, data collection year, study design, sampling frame, recruitment setting, sample size, number of cases and controls where applicable, participant age range or mean age, sex distribution, and follow-up duration for longitudinal studies.

Exposure data included the absenteeism construct, measurement source, reference period, exposure threshold, comparison group, and whether absenteeism was measured by self-report, school records, linked administrative data, or another source. Outcome data included the type of outcome, outcome definition, measurement instrument or data source, reference period, and whether the outcome was self-reported, clinically assessed, recorded in hospital or emergency department data, or identified from mortality records.

For each relevant exposure-outcome association, the extracted quantitative data included the reported effect estimate, 95% confidence interval, standard error, *p* value, raw cell counts when available, and the covariates included in adjusted models. Adjusted estimates were prioritised over unadjusted estimates when both were available. When several adjusted models were reported, the model with the most complete adjustment for prespecified confounders was extracted, provided it did not clearly adjust only for variables judged to be downstream consequences of absenteeism. If only unadjusted estimates or raw data were available, these were extracted and flagged for sensitivity analysis or narrative synthesis.

When a study reported multiple absenteeism categories, the estimate comparing the highest or most clearly defined absenteeism category with the lowest or no-absence reference category was extracted for the main analysis, unless an overall binary estimate was available. When studies reported sex-specific, age-specific, country-specific, or other subgroup estimates without an overall estimate, subgroup estimates were extracted separately only when the groups were non-overlapping; otherwise, estimates were combined where possible before inclusion in the main meta-analysis.

To reduce the risk of double-counting participants, included studies were checked for potential dataset overlap by country, survey system, survey year or wave, sampling frame, age range, exposure definition, outcome definition, and authorship. Where multiple publications appeared to use overlapping datasets, priority was given to the estimate with the largest relevant sample size, the clearest attendance-related exposure definition, and the most appropriate adjustment for confounding. A study-level dataset overlap assessment is provided in Supplementary Table S7, and sensitivity analyses excluding potentially overlapping pooled-country estimates are provided in Supplementary Table S8.

Data extraction was completed by one reviewer and checked by a second reviewer. Discrepancies were resolved by reference to the full text and, where necessary, by consensus. All extracted effect estimates were coded so that values greater than 1 indicated higher odds or risk of self-harm or suicidal outcomes among participants with greater absenteeism. The study-specific estimates included in each meta-analysis are reported in Supplementary Tables S3–S5.

### 2.7. Risk of Bias Assessment

Study-level risk of bias was evaluated using a modified ROBINS-I framework adapted for observational exposure studies. The following domains were assessed: bias due to confounding, bias in selection of participants, bias in classification of exposure, bias due to deviations from the intended exposure, bias due to missing data, bias in measurement of outcomes, and bias in selection of the reported result.

Because the exposure of interest was school absenteeism rather than an assigned intervention, ROBINS-I was not applied as an intervention-specific tool without modification. Instead, its domains were adapted to the context of observational exposure studies. The assessment focused on whether each study provided an internally valid estimate of the association between attendance-related exposure and self-harm or suicidal outcomes. Particular emphasis was placed on confounding, participant selection, clarity and timing of exposure classification, missing data, outcome measurement, and selective reporting. The domain concerning deviations from intended interventions was interpreted as timing-related or post-exposure bias, including whether changes in attendance status, overlapping exposure-outcome measurement windows, or post-exposure factors could have affected the estimate. Study-level domain judgements are provided in Supplementary Table S6.

For confounding, particular attention was given to whether studies accounted for major demographic, family, school, behavioral, and mental health factors that could plausibly influence both absenteeism and self-harm or suicidal outcomes. These included age, sex or gender, socioeconomic position, family structure or parental support, bullying or violence exposure, substance use, mental health symptoms, prior self-harm or suicidality, and school-related factors, where relevant to the study design. For selection bias, assessments considered sampling frame, participation rate, exclusions, attrition, and whether the analytic sample was representative of the target school-aged population.

Exposure classification was judged according to the clarity, validity, timing, and source of the absenteeism measure. Outcome measurement was judged according to the definition, ascertainment method, reference period, and likely misclassification of self-harm or suicidal outcomes. Missing data judgements considered the extent of missing exposure, outcome, and covariate data and whether appropriate methods were used to address missingness. Selective reporting was assessed by comparing reported methods, tables, Supplementary Material, and results to determine whether relevant absenteeism-specific findings appeared to be incompletely reported.

Each domain was rated as low, moderate, serious, or critical risk of bias, with an overall judgement assigned according to the highest level of concern across domains. Risk of bias assessments were completed by one reviewer and checked by a second reviewer. Disagreements were resolved through discussion and, where necessary, by consultation with a third reviewer.

The signalling questions used to guide the adapted risk-of-bias assessment are described in the Supplementary Material, and study-level domain judgements are provided in Supplementary Table S6.

### 2.8. Data Synthesis and Meta-Analysis

Findings were initially summarised narratively according to outcome domain, study design, exposure definition, and data source. Meta-analysis was conducted when at least three sufficiently comparable estimates were available for the same outcome category. Separate meta-analyses were conducted for suicidal ideation, suicide plan, and suicide attempt. Studies of non-suicidal self-injury, deliberate self-harm, suicide death, or composite suicidal behaviour were synthesised narratively when outcome definitions, exposure definitions, or effect measures were too heterogeneous for pooling.

The primary effect measure for quantitative synthesis was the odds ratio (OR). Reported ORs and 95% confidence intervals were transformed to the log scale before pooling. Standard errors were calculated from confidence intervals using the formula SE = (ln upper CI − ln lower CI)/3.92. When studies reported sufficient two-by-two data, crude ORs and corresponding confidence intervals were calculated. Effect estimates reported in the opposite direction were inverted so that all estimates reflected the association between greater absenteeism and higher odds of the outcome. Risk ratios or hazard ratios were not pooled with odds ratios unless an odds ratio could be calculated from the available data or the estimate was judged sufficiently comparable for the planned synthesis; otherwise, such findings were described narratively.

Pooled estimates were calculated using inverse-variance methods. Both common-effect and random-effects models were estimated, with the DerSimonian–Laird method used for the random-effects model. Because included studies were expected to vary by country, survey system, study design, exposure threshold, outcome definition, and confounder adjustment, random-effects estimates were prioritised for interpretation when heterogeneity was present. Pooled log estimates were exponentiated and presented as odds ratios (ORs) with 95% confidence intervals.

Between-study heterogeneity was assessed using Cochran’s Q test, the between-study variance (tau-squared), and the I-squared statistic. I-squared values were interpreted as indicating low, moderate, or substantial heterogeneity using conventional thresholds, while recognising that interpretation depends on the number of studies and precision of individual estimates. Funnel plots were generated to visually assess small-study effects and potential publication bias; formal tests for funnel plot asymmetry were interpreted cautiously and were not emphasised when fewer than ten estimates were available for an outcome.

Where data permitted, subgroup analyses were planned according to exposure type, outcome type, study design, age group, sex or gender, geographical region, data source, and whether estimates were adjusted for confounders. Because quantitative syntheses were predominantly based on truancy, skipped school, or unauthorised absence, subgroup and narrative interpretations distinguished these constructs from broader attendance-related measures such as chronic or persistent absence, school refusal or avoidance, and absence because of feeling unsafe. Sensitivity analyses examined alternative pooling assumptions, including common-effect models, DerSimonian–Laird random-effects models, REML random-effects models, Hartung–Knapp-type confidence intervals, prediction intervals, and independent-estimate analyses excluding potentially overlapping pooled-country estimates. Numerical subgroup and sensitivity results are provided in Supplementary Table S8.

All statistical tests were two-sided, and statistical significance was evaluated at *p* < 0.05. Analyses were conducted using R version 4.5.1 with the meta and metafor packages.

### 2.9. Certainty-of-Evidence Assessment

For each pooled outcome, certainty of evidence was judged using an adapted GRADE approach for observational evidence. The assessment considered risk of bias, inconsistency, indirectness, imprecision, and reporting or publication bias. Because all included studies were observational and many were cross-sectional, certainty ratings were interpreted cautiously. Risk of bias was considered in relation to the distribution of study-level ratings and the absence of studies judged to have low overall risk of bias. Inconsistency was assessed using the magnitude and direction of effects, I-squared values, and overlap of confidence intervals. Indirectness was considered in relation to population, exposure definition, outcome definition, and applicability to school-aged children and adolescents. Imprecision was assessed using the width of confidence intervals, number of estimates, and stability of pooled estimates across sensitivity analyses. Reporting or publication bias was considered using funnel plots where appropriate and by considering selective availability of attendance-specific estimates. Certainty was rated separately for suicidal ideation, suicide plan, and suicide attempt as high, moderate, low, or very low. Domain-level certainty-of-evidence judgements for each pooled outcome are provided in Supplementary Table S10.

## 3. Results

### 3.1. Study Selection

Database searches identified 1189 records: 39 from PubMed, 491 from Web of Science Core Collection, and 659 from Scopus. After removal of 411 duplicate records, 778 records remained and were screened by title and abstract. Of these, 754 records were excluded because they did not meet the eligibility criteria.

Twenty-four reports were selected for full-text retrieval. One report could not be retrieved; therefore, 23 full-text reports were assessed for eligibility. After full-text assessment, 8 reports were excluded. The reasons for exclusion were: wrong exposure, where school absenteeism was not the main exposure (n = 3); no extractable absenteeism-specific effect estimate suitable for quantitative synthesis (n = 3); and wrong population, mainly clinical or inpatient samples rather than school-enrolled general populations (n = 2). The full list of excluded full-text reports, with citations and specific reasons for exclusion, is provided in Supplementary Table S9.

The final review included 47 studies: 15 newly identified through the updated search and 32 retained from the previous review after reassessment against the current eligibility criteria. Figure 1 presents the updated-search pathway; the previous-review contribution is described in text.

### 3.2. Study Characteristics

The review included 47 studies examining associations between school attendance-related factors and suicidal or self-harm outcomes among children, adolescents, and young people. The studies were conducted across a wide range of geographical settings, including North America, Europe, Asia, Africa, Latin America, and Oceania. The USA was the most frequent study setting, while other studies were conducted in countries such as China, Benin, New Zealand, the UK/Wales, France, Ghana, Swaziland, Malaysia, Mongolia, Turkey, India, Peru, Nepal, Tonga, Samoa, Bolivia, and Argentina. Several studies also used multinational or pooled survey datasets.

Most included studies used cross-sectional school-based survey designs or secondary analyses of large population datasets, such as the Global School-based Student Health Survey, Youth Risk Behavior Surveillance System, ESPAD, SEYLE, and Add Health. A smaller number of studies used prospective cohort, retrospective record-linkage, matched case–control, or nested case–control designs. More recent studies increasingly used linked administrative education and health records.

Sample sizes varied substantially, ranging from small clinical or community samples of adolescents who had attempted suicide to large population-based record-linkage cohorts including more than 500,000 pupils. The study populations were mainly school-aged adolescents, commonly between 10 and 18 years, although some studies included older youth up to 24 years or followed participants from childhood into adolescence or young adulthood. A small number of studies included young people older than 18 years. These studies were retained because the exposure related to school, college, or education attendance, but their inclusion was examined in sensitivity analysis where data permitted. Where reported, the proportion of female participants was generally close to half of the sample, although some clinical and case–control studies included a higher proportion of females.

School attendance-related exposures were described using terms such as truancy, absenteeism, missed classes, skipped school, poor attendance, persistent absence, school exclusion, and absence because of feeling unsafe at school. Covariate adjustment varied across studies, but commonly included age, sex, socioeconomic indicators, parental support or monitoring, peer relationships, bullying and violence exposure, substance use, mental health symptoms, previous suicidal behaviour, and wider school-related factors.

The estimates included in the quantitative syntheses were predominantly based on truancy, skipped school, or unauthorised absence. Evidence for broader constructs, including chronic or persistent absence, school refusal or avoidance, absence due to feeling unsafe, and administratively recorded poor attendance, was more limited and was interpreted separately in subgroup, sensitivity, or narrative synthesis.

Overall, the included evidence base was heterogeneous in terms of study design, country setting, sample size, exposure definition, outcome measurement, and confounder adjustment. However, the literature was dominated by cross-sectional school-based studies, with fewer longitudinal and administrative record-linkage studies available.

The dataset overlap assessment identified potential overlap between the pooled West African GSHS analysis by Diallo et al. [23] and country-specific GSHS analyses from Ghana and Benin. To assess the influence of this potential dependency, sensitivity analyses were conducted excluding Diallo et al. from the suicidal ideation and suicide attempt syntheses. The direction of effect was unchanged, and the pooled estimates remained above the null. No potential overlapping estimate was identified for the suicide plan synthesis.

Detailed characteristics of the included studies are presented in Table 1.

### 3.3. Risk of Bias Results

The risk of bias assessment included 47 studies. Overall, 26 studies (55%) were judged to have a moderate risk of bias, while 21 studies (45%) were rated as having a serious risk of bias. No studies were assessed as having a low overall risk of bias or a critical overall risk of bias.

The main sources of bias were confounding and selection bias. For confounding, 28 studies (60%) were rated as moderate risk and 19 studies (40%) as serious risk. For selection bias, 8 studies (17%) were rated as low risk, 31 studies (66%) as moderate risk, and 8 studies (17%) as serious risk. Exposure classification was also predominantly rated as moderate risk, with 37 studies (79%) falling into this category, while 4 studies (9%) were low risk and 6 studies (13%) were serious risk.

All studies were rated as low risk for deviations from the intended exposure, indicating that this domain was not a major contributor to overall bias. For missing data, 17 studies (36%) were judged as low risk, 29 studies (62%) as moderate risk, and 1 study (2%) as serious risk. Outcome measurement was mostly rated as moderate risk, with 36 studies (77%) in this category, 7 studies (15%) at low risk, and 4 studies (9%) at serious risk. Selective reporting showed a relatively balanced distribution, with 24 studies (51%) rated as low risk and 23 studies (49%) as moderate risk.

In summary, most included studies had either moderate or serious overall risk of bias. The overall judgement was mainly driven by concerns related to confounding, selection bias, and exposure classification, whereas deviations from intended exposure and selective reporting were less problematic.

Study-level domain judgements and reasons for overall risk-of-bias ratings are provided in Supplementary Table S6. Figure 2 presents the study-level risk-of-bias assessment.

### 3.4. Meta-Analysis: Association Between School Absenteeism and Suicidal Ideation

Twelve study estimates were included in the meta-analysis of suicidal ideation. The common-effect model showed a pooled odds ratio (OR) of 1.26 (95% CI: 1.22–1.31), while the DerSimonian–Laird random-effects model showed a pooled OR of 1.32 (95% CI: 1.19–1.46). This indicates that the exposure was associated with significantly increased odds of suicidal ideation, corresponding to approximately 32% higher odds in the random-effects model. Between-study heterogeneity was moderate to substantial (I^2^ = 63.3%, τ^2^ = 0.0148, Q = 29.98, df = 11, *p* = 0.0016), supporting the use of the random-effects estimate for interpretation. Visual inspection of the funnel plot was performed to assess possible small-study effects or publication bias; some dispersion of estimates was observed, but interpretation should be cautious. Overall, the findings suggest a statistically significant association with increased odds of suicidal ideation (Figure 3).

### 3.5. Meta-Analysis: Association Between School Absenteeism and Suicide Plan

Six study estimates were included in the meta-analysis of suicide plan. The pooled effect showed significantly increased odds of suicide plan, with both the common-effect and DerSimonian–Laird random-effects models producing an identical estimate (OR = 1.31, 95% CI: 1.21–1.42). This indicates that the exposure was associated with approximately 31% higher odds of suicide plan. There was no evidence of meaningful between-study heterogeneity (I^2^ = 0.0%, τ^2^ = 0.0000, Q = 4.23, df = 5, *p* = 0.5171), suggesting that the study estimates were highly consistent. Visual inspection of the funnel plot was used to assess possible small-study effects or publication bias; however, interpretation should be cautious because of the limited number of included estimates. The funnel plot includes the same six estimates as the forest plot and Supplementary Table S4. Overall, the findings indicate a statistically significant association with suicide plan (Figure 4).

### 3.6. Meta-Analysis: Association Between School Absenteeism and Suicide Attempt

Fourteen study estimates were included in the meta-analysis of suicide attempt. The common-effect model showed a pooled odds ratio (OR) of 1.52 (95% CI: 1.44–1.60), while the DerSimonian–Laird random-effects model showed a pooled OR of 1.60 (95% CI: 1.41–1.81). This indicates that the exposure was associated with significantly increased odds of suicide attempt, corresponding to approximately 60% higher odds in the random-effects model. Moderate to substantial between-study heterogeneity was observed (I^2^ = 69.4%, τ^2^ = 0.0318, Q = 42.42, df = 13, *p* < 0.001), supporting interpretation of the random-effects estimate. Visual inspection of the funnel plot was conducted to assess possible small-study effects or publication bias; some dispersion of estimates was present, but this should be interpreted cautiously. Overall, the findings suggest a statistically significant association with increased odds of suicide attempt (Figure 5).

### 3.7. Narrative Synthesis of Self-Harm, Non-Suicidal Self-Injury, Composite Suicidal Behaviour, and Suicide Death

Outcomes that were not suitable for quantitative pooling were synthesised narratively. These included non-suicidal self-injury, deliberate self-harm, composite suicidal behaviour outcomes, and suicide death. Pooling was not conducted for these outcomes because definitions, outcome sources, effect measures, exposure definitions, and study designs varied substantially across studies.

Studies of non-suicidal self-injury and deliberate self-harm generally suggested that attendance-related problems may co-occur with broader psychosocial vulnerability, including bullying, low school connectedness, emotional distress, and unsafe school environments. However, the measures used across studies were heterogeneous and did not provide sufficiently comparable estimates for meta-analysis.

Studies reporting composite suicidal behaviour outcomes combined ideation, planning, attempts, or broader suicidal behaviour measures. These outcomes were not pooled because they were not directly comparable with the outcome-specific meta-analyses of suicidal ideation, suicide plan, and suicide attempt.

Studies of suicide death or hospital-recorded self-harm provided clinically important evidence but were not pooled because these outcomes were rarer, measured using different data sources, and reported different effect measures. Overall, the narrative evidence was broadly consistent with the pooled findings, but certainty remained limited by observational design, heterogeneous measurement, and risk of bias.

### 3.8. Subgroup Analyses

Subgroup analyses were conducted to explore potential sources of between-study heterogeneity and to assess whether pooled associations varied by exposure definition, study design, data source, and adjustment level. Planned subgroup analyses by age group, sex/gender, and geographical region were also assessed for feasibility but were not pooled when fewer than three sufficiently comparable estimates were available. These analyses were exploratory because the number of estimates was limited in several strata, and exposure definitions varied across studies.

The quantitative syntheses were mainly informed by estimates for truancy, skipped school, or unauthorised absence. Findings for broader attendance constructs, including chronic or persistent absence, school refusal or avoidance, and absence due to feeling unsafe, were less consistently available and were therefore interpreted as supporting contextual evidence rather than as fully comparable pooled effects.

Patterns across study designs and adjustment strata were therefore interpreted as hypothesis-generating rather than confirmatory. Cross-sectional school-based surveys contributed most of the pooled evidence, while longitudinal, record-linkage, and case–control studies were fewer and less directly comparable.

Numerical sensitivity results and subgroup-analysis feasibility statements are provided in Supplementary Table S8. Where at least three sufficiently comparable estimates were available within a stratum, subgroup pooled estimates were calculated. Where fewer than three comparable estimates were available, the subgroup was explicitly labelled as not pooled. These analyses did not identify a single study-level characteristic that fully explained heterogeneity for suicidal ideation or suicide attempt.

Across all subgroup analyses, findings should be interpreted cautiously because exposure thresholds, reference periods, data sources, and confounder adjustment sets differed substantially across studies.

### 3.9. Sensitivity Analyses

Sensitivity analyses were used to assess whether the pooled findings were sensitive to dataset dependency, pooling assumptions, and random-effects model specification. Numerical results were generated from the same final analytical dataset used for Supplementary Tables S3–S5 and Figure 3, Figure 4 and Figure 5 and are provided in Supplementary Table S8.

The direction of effect remained consistent when comparing common-effect and DerSimonian–Laird random-effects models. Additional REML random-effects models with Hartung–Knapp-type confidence intervals also supported the same direction of association: suicidal ideation, OR = 1.33 (95% CI: 1.16–1.53); suicide plan, OR = 1.31 (95% CI: 1.19–1.45); and suicide attempt, OR = 1.64 (95% CI: 1.36–1.99). Prediction intervals were wider and crossed the null for suicidal ideation and suicide attempt, indicating that effects may vary across future populations.

Independent-estimate sensitivity analyses excluding Diallo et al. [23], which potentially overlapped with country-specific Ghana and Benin GSHS estimates, did not materially change the direction of findings. The DerSimonian–Laird pooled OR changed from 1.32 to 1.29 for suicidal ideation and from 1.60 to 1.63 for suicide attempt. No overlap-related exclusion was required for suicide plan. These analyses reduce, but do not eliminate, concerns about dependency, residual confounding, exposure misclassification, selection bias, and outcome measurement bias.

### 3.10. Certainty of Evidence

The certainty of evidence was rated as low for all three pooled outcomes. This judgement reflected the observational nature of the evidence base, the predominance of cross-sectional school-based surveys, the absence of studies judged to have low overall risk of bias, residual confounding, potential reverse causation, and heterogeneity in exposure and outcome measurement. Certainty was not rated as high or moderate for any pooled outcome.

A summary of pooled findings and certainty of evidence is presented in Table 2. Domain-level certainty-of-evidence judgements are provided in Supplementary Table S10.

## 4. Discussion

### 4.1. Main Findings

This systematic review and meta-analysis synthesised evidence from 47 studies examining the association between attendance-related problems and self-harm or suicidal outcomes among children, adolescents, and school-aged young people. The findings provide low-certainty observational evidence that attendance-related constructs are associated with increased odds of suicidal ideation, suicide plan, and suicide attempt. The quantitative syntheses were predominantly based on truancy, skipped school, or unauthorised absence, rather than on chronic absence, persistent absence, or school refusal.

The largest pooled association was observed for suicide attempt. This may indicate a stronger association with more severe suicidal outcomes, but this interpretation should remain cautious because the included studies differed in exposure definition, outcome measurement, adjustment for confounding, study design, and potential dataset dependency. Suicidal ideation and suicide attempt showed moderate-to-substantial heterogeneity, whereas suicide plan showed no meaningful heterogeneity.

The subgroup and narrative findings support a distinction between different attendance constructs. The meta-analytic evidence was strongest for truancy, skipped school, or unauthorised absence. Evidence for chronic or persistent absence, school refusal or avoidance, administratively recorded poor attendance, and absence because of feeling unsafe was more limited and should not be interpreted as equivalent to the pooled estimates.

Sensitivity analyses addressing potential dataset overlap and alternative random-effects specifications did not change the direction of association, although prediction intervals indicated uncertainty about the range of effects expected in future populations. The evidence base remained limited by risk of bias: more than half of the studies were judged to have moderate risk of bias, almost half were judged to have serious risk of bias, and no study was rated as having low overall risk of bias. Therefore, the findings support an association between attendance-related problems and suicidal outcomes but do not establish that absenteeism is a direct causal risk factor.

### 4.2. Comparison with Previous Studies

The findings are consistent with previous adolescent mental health research showing that school-related difficulties are associated with suicidal thoughts, self-harm, and suicide attempts. Earlier school-based studies have identified truancy, poor school connectedness, low academic engagement, unsafe school environments, bullying, substance use, and family adversity as correlates of suicidal behaviour among adolescents [5,6,7,9,10,11,12,13,14,15]. The present review extends this literature by focusing specifically on absenteeism and by quantitatively pooling associations for suicidal ideation, suicide plan, and suicide attempt.

The results also align with longitudinal and record-linkage studies suggesting that attendance problems may precede later self-harm or suicide-related outcomes. For example, prospective cohort and administrative record studies from New Zealand, Wales, and the USA have reported associations between truancy, persistent absence, or school disengagement and later suicidal behaviour, self-harm presentations, or suicide mortality [7,17,18,19,21,22]. These studies are particularly important because they reduce, although do not eliminate, concerns about reverse causation.

At the same time, the review highlights important differences across the literature. Many cross-sectional studies measured absenteeism and suicidal outcomes over similar or overlapping timeframes, making it difficult to determine whether absenteeism preceded suicidality, followed it, or occurred as part of the same underlying episode of distress. Some studies measured absenteeism as truancy or skipped school, while others used administrative indicators of persistent absence or absence due to feeling unsafe at school. These constructs are related but not identical. Truancy may reflect rule-breaking, disengagement, peer influence, or lack of supervision, whereas school refusal or avoidance may be more closely linked to anxiety, depression, bullying, or perceived threat. Persistent absence captured through school records may also include illness, family circumstances, exclusionary processes, or unmet educational needs. Therefore, evidence for these constructs was distinguished in interpretation rather than treated as fully interchangeable.

The current findings are also consistent with studies of non-suicidal self-injury and deliberate self-harm, although these outcomes were less suitable for pooled analysis because of variation in definitions, measurement methods, and clinical severity. Studies of self-injury and self-harm suggest that school safety, peer relationships, bullying, emotional distress, and school connectedness may be important correlates of self-injurious behaviour [3,8,20,30,39]. This supports the interpretation that absenteeism may be part of a wider pattern of school and psychosocial vulnerability rather than an isolated behavioural exposure.

### 4.3. Possible Mechanisms

Several mechanisms may explain the observed association between school absenteeism and suicidal outcomes. First, absenteeism may be an early indicator of underlying mental health problems. Depression, anxiety, trauma symptoms, substance use, neurodevelopmental difficulties, and behavioural problems can all impair school attendance. In this pathway, absenteeism may not directly cause suicidality, but may signal the presence of emotional distress or functional impairment that also increases suicide risk [4,7,8,14,19,20,30].

Second, school absenteeism may contribute to suicidal outcomes through social disconnection. Regular school attendance provides access to peer relationships, trusted adults, routine, structure, and opportunities for help-seeking. Repeated absence may weaken these protective connections and increase isolation. For young people already experiencing distress, reduced contact with school staff and peers may decrease the likelihood that warning signs are identified and support is offered [2,3,4,14].

Third, absenteeism may reflect exposure to adverse school environments. Some included studies measured absence because of feeling unsafe at school, while others adjusted for bullying, violence, weapon carrying, fights, or peer victimisation. In these contexts, absenteeism may be a behavioural response to threat, humiliation, exclusion, or fear. Bullying and school violence are established correlates of adolescent suicidality, and absenteeism may lie on the pathway between victimisation and suicidal outcomes or may act as a visible marker of ongoing exposure to harm [3,4,14,16,50].

Fourth, absenteeism may increase academic difficulties and perceived failure. Frequent absence can disrupt learning, reduce academic attainment, increase conflict with teachers or parents, and contribute to disciplinary involvement. These consequences may intensify hopelessness, low self-worth, or perceived burdensomeness, particularly among adolescents with pre-existing mental health problems or limited family support [4,14,19].

Fifth, absenteeism may be linked to broader family and social adversity. Food insecurity, poverty, family instability, weak parental monitoring, maltreatment, substance use, and community violence were commonly included as covariates across the included studies. These factors may increase both the likelihood of absence and the likelihood of suicidal outcomes. The persistence of positive associations in adjusted analyses suggests that measured confounding does not fully explain the relationship, but residual confounding remains likely [5,6,7,8,9,10,11,12,13,14,15].

Finally, the association may be bidirectional. Suicidal ideation, self-harm, and suicide attempts may lead to absence through emotional distress, hospitalisation, stigma, family disruption, or school avoidance after a crisis. At the same time, absenteeism may exacerbate vulnerability by reducing social support and increasing academic or disciplinary problems. Future longitudinal studies with repeated measures are needed to separate these temporal pathways [4,17,18,19,21,22].

### 4.4. Clinical and School Implications

These findings have important implications for schools, clinicians, public health services, and child and adolescent mental health systems. School absenteeism is routinely recorded in many education systems and may therefore provide a practical early-warning indicator of psychosocial risk. The results suggest that students with frequent, persistent, or unexplained absences should not be viewed only through an attendance-compliance or disciplinary lens. Instead, absenteeism should prompt a supportive assessment of wellbeing, safety, family circumstances, bullying, mental health symptoms, and self-harm or suicide risk [2,4].

Schools should consider integrating attendance monitoring with pastoral care, safeguarding, and mental health support. Patterns such as sudden increases in absence, repeated unauthorised absence, chronic absence, or absence linked to feeling unsafe may warrant timely review by school counsellors, nurses, safeguarding leads, or multidisciplinary support teams. However, the response should be non-punitive. Punitive attendance policies may worsen disengagement, conceal underlying distress, or disproportionately affect students experiencing poverty, disability, neurodevelopmental conditions, caregiving responsibilities, bullying, or family adversity [2,3,4].

Different attendance-related constructs may require different responses. Truancy or unauthorised absence may indicate disengagement, peer problems, family adversity, weak supervision, or risk-taking contexts. Chronic or persistent absence may reflect cumulative educational, social, family, or health-related difficulties. School refusal or avoidance may be more closely linked to anxiety, fear, bullying, trauma, or perceived lack of safety. Absence because of feeling unsafe may point to school violence, victimisation, or poor school connectedness. Therefore, attendance problems should be assessed in context rather than treated as a single homogeneous exposure [3,4,50]. For clinicians, school attendance should be routinely explored when assessing children and adolescents with depression, anxiety, self-harm, suicidal ideation, or behavioural problems. Attendance difficulties may provide information about functional impairment, peer difficulties, family stress, sleep disturbance, substance use, trauma exposure, or school avoidance. Conversely, when young people present with persistent absenteeism, clinicians and school health professionals should consider screening for mood symptoms, anxiety, bullying, self-harm, and suicidal thoughts [2,4,14].

The findings also support closer collaboration between education, primary care, mental health, and social services. Attendance data alone should not be used as a suicide risk prediction tool, because the absolute risk of suicidal behaviour among absent students will vary substantially and many absent students will not be suicidal. Nevertheless, when combined with contextual information, attendance problems may help identify young people who require earlier support. This is especially relevant because some adolescents who are at risk of self-harm or suicide may not actively seek help.

At the policy level, interventions aimed at improving school attendance should be linked with mental health promotion, anti-bullying strategies, trauma-informed school practice, family support, and accessible referral pathways. Efforts to improve attendance are more likely to be effective and ethical when they address the reasons for absence rather than focusing only on enforcement [2,3,4,14,16].

### 4.5. Strengths and Limitations

This review has several strengths. It used a broad eligibility framework that captured multiple forms of school absenteeism and a range of self-harm and suicidal outcomes. The search included multiple major databases and incorporated studies from a previous review as well as newly identified studies. The evidence base was geographically diverse, including studies from North America, Europe, Asia, Africa, Latin America, and Oceania. The review also included a wide range of study designs, from cross-sectional school surveys to prospective cohorts and large administrative record-linkage studies.

Another strength is that separate meta-analyses were conducted for suicidal ideation, suicide plan, and suicide attempt. This is important because these outcomes differ in prevalence, severity, and clinical meaning. The review also assessed risk of bias systematically and conducted subgroup and sensitivity analyses to examine the robustness of the findings. The consistency of positive associations across outcomes and analytic assumptions strengthens confidence in the overall conclusion.

Limitations should be considered at two levels: limitations of the included studies and limitations of the review process. At the level of included studies, the evidence base was limited by the predominance of observational and cross-sectional designs, reliance on self-reported attendance and suicidal outcomes in many studies, residual confounding, variation in adjustment sets, and the absence of studies judged to have low overall risk of bias. At the review level, limitations included heterogeneity in exposure and outcome definitions, restriction to studies available in English, omission of grey literature, and search-related limitations. First, most included studies were observational, and many were cross-sectional. Therefore, causal inference is limited. In cross-sectional studies, absenteeism and suicidal outcomes may have occurred during the same period, making it difficult to establish temporal order. Reverse causation is plausible, especially where suicidal ideation, self-harm, depression, or anxiety may have contributed to school absence. Therefore, the observed associations should be interpreted as bidirectional markers of vulnerability rather than evidence that absenteeism is a direct causal determinant of suicidal outcomes.

Second, residual confounding is likely. Although many studies adjusted for demographic, family, psychosocial, behavioural, and school-related factors, adjustment sets varied considerably. Some studies did not adjust for key confounders such as prior mental health problems, previous self-harm or suicidality, bullying, trauma exposure, family adversity, substance use, neurodevelopmental conditions, or socioeconomic disadvantage. Because no included study was rated as low overall risk of bias, the pooled estimates should be interpreted as associations rather than causal effects.

Third, the exposure definition varied across studies. The quantitative syntheses were predominantly based on truancy, skipped school, or unauthorised absence, whereas evidence for chronic absence, persistent absence, school refusal, school avoidance, administratively recorded poor attendance, and absence because of feeling unsafe was more limited. These constructs may reflect different underlying processes and should not be interpreted as identical. Differences in reference periods, thresholds, and measurement sources may have contributed to heterogeneity, especially for suicidal ideation and suicide attempt.

Fourth, outcome measurement also varied. Many studies relied on self-reported suicidal ideation, suicide plans, or suicide attempts, often using single survey items. Others used clinical, hospital, emergency department, administrative, or mortality data. Self-report may be affected by recall bias, stigma, misunderstanding of survey items, or under-reporting. Administrative and clinical records may capture more severe outcomes but miss young people who do not present to services.

Fifth, the meta-analyses pooled odds ratios. For outcomes that are not rare, odds ratios may overestimate risk ratios. Therefore, the pooled estimates should be interpreted as relative odds, not direct estimates of absolute risk. The public health significance of the findings depends not only on the relative association but also on the baseline prevalence of absenteeism and suicidal outcomes in each population.

Sixth, although funnel plots were inspected, formal assessment of publication bias was limited by the number of studies available for some outcomes. For suicide plan, only six estimates were included, making small-study effects difficult to evaluate. In addition, studies that measured absenteeism but did not report absenteeism-specific estimates may have been missed or excluded because relevant data were not extractable.

Finally, the included studies largely focused on school-enrolled adolescents. Young people who had already dropped out of school, were excluded from mainstream education, were in alternative education settings, or were not captured by school surveys may be underrepresented. These groups may have particularly high levels of both absenteeism-related vulnerability and suicide risk.

In addition, the updated search was limited to PubMed/MEDLINE, Web of Science Core Collection, and Scopus. The omission of PsycINFO, Embase, ERIC, CINAHL, and Education Source was reconsidered during revision and remains a limitation, because eligible studies from psychology, psychiatry, education, nursing, and allied health studies may have been missed. Earlier coverage was partly supplemented by the previous review, which searched MEDLINE, PsycINFO, Embase, ERIC, and the British Education Index up to 6 June 2018 [4], but the updated search did not repeat these discipline-specific databases.

In addition, full-text eligibility was limited to studies available in English. This language restriction may have introduced language bias and may have led to the omission of eligible studies published in other languages.

### 4.6. Future Research

Future research should prioritise longitudinal designs with repeated measures of attendance, mental health, self-harm, and suicidal outcomes. Such studies are needed to clarify whether absenteeism precedes suicidality, follows it, or operates bidirectionally. Studies should also examine dose–response associations, including whether risk increases with frequency, duration, persistence, or escalation of absence.

Greater standardisation of exposure definitions is needed. Future studies should distinguish between authorised absence, unauthorised absence, truancy, school refusal, illness-related absence, exclusion-related absence, chronic absence, and absence due to feeling unsafe. These categories may have different causes and different implications for intervention. Administrative attendance records should be linked, where possible, with survey or clinical data to capture both objective attendance patterns and the young person’s reasons for absence.

Research should also examine mechanisms and moderators. Important areas include bullying, peer isolation, school connectedness, academic pressure, family adversity, socioeconomic disadvantage, neurodevelopmental conditions, substance use, depression, anxiety, trauma, and prior self-harm. Mediation analyses and causal modelling approaches could help determine whether absenteeism is an independent risk factor, a mediator, a consequence, or a marker of wider vulnerability.

More evidence is needed from low- and middle-income countries, alternative education settings, rural areas, and marginalised groups. Future studies should examine whether associations differ by sex or gender, age, socioeconomic position, ethnicity, disability, neurodevelopmental status, sexual orientation, gender identity, and exposure to violence or displacement. These analyses are important because both absenteeism and suicide risk are shaped by structural and social determinants.

Finally, intervention studies are needed to determine whether addressing absenteeism can reduce self-harm or suicidal outcomes. Attendance interventions should be evaluated not only for their effect on school attendance, but also for their impact on mental health, perceived safety, family support, bullying, help-seeking, and self-harm. Interventions are likely to be most effective when they are supportive, trauma-informed, and integrated with school mental health services rather than based primarily on sanctions or enforcement.

Overall, future research should move beyond documenting association and toward identifying which types of absenteeism matter most, which young people are most at risk, what mechanisms explain the association, and which interventions can reduce both absence and suicide-related harm.

## 5. Conclusions

This systematic review and meta-analysis found low-certainty observational evidence that attendance-related problems, particularly truancy, skipped school, or unauthorised absence, are associated with increased odds of suicidal ideation, suicide plan, and suicide attempt among children, adolescents, and school-aged young people. Across 47 included studies, the primary random-effects meta-analyses showed 32% higher odds of suicidal ideation, 31% higher odds of suicide plan, and 60% higher odds of suicide attempt.

Absenteeism may be considered one potential marker of psychosocial vulnerability, particularly when combined with contextual information about mental health, bullying, family adversity, school safety, social support, and school connectedness. However, absenteeism should not be interpreted as a standalone suicide risk prediction tool, and the findings do not establish a direct causal relationship. Evidence for chronic absence, persistent absence, school refusal, and school avoidance remains more limited than evidence for truancy-related measures.

The evidence base was limited by observational and cross-sectional designs, residual confounding, reverse causation, heterogeneous exposure and outcome definitions, and the absence of studies judged to have low overall risk of bias. Future research should prioritise longitudinal designs, standardised absenteeism definitions, dose–response analyses, and evaluation of supportive, non-punitive responses to attendance difficulties.

## Figures and Tables

**Figure 1:**
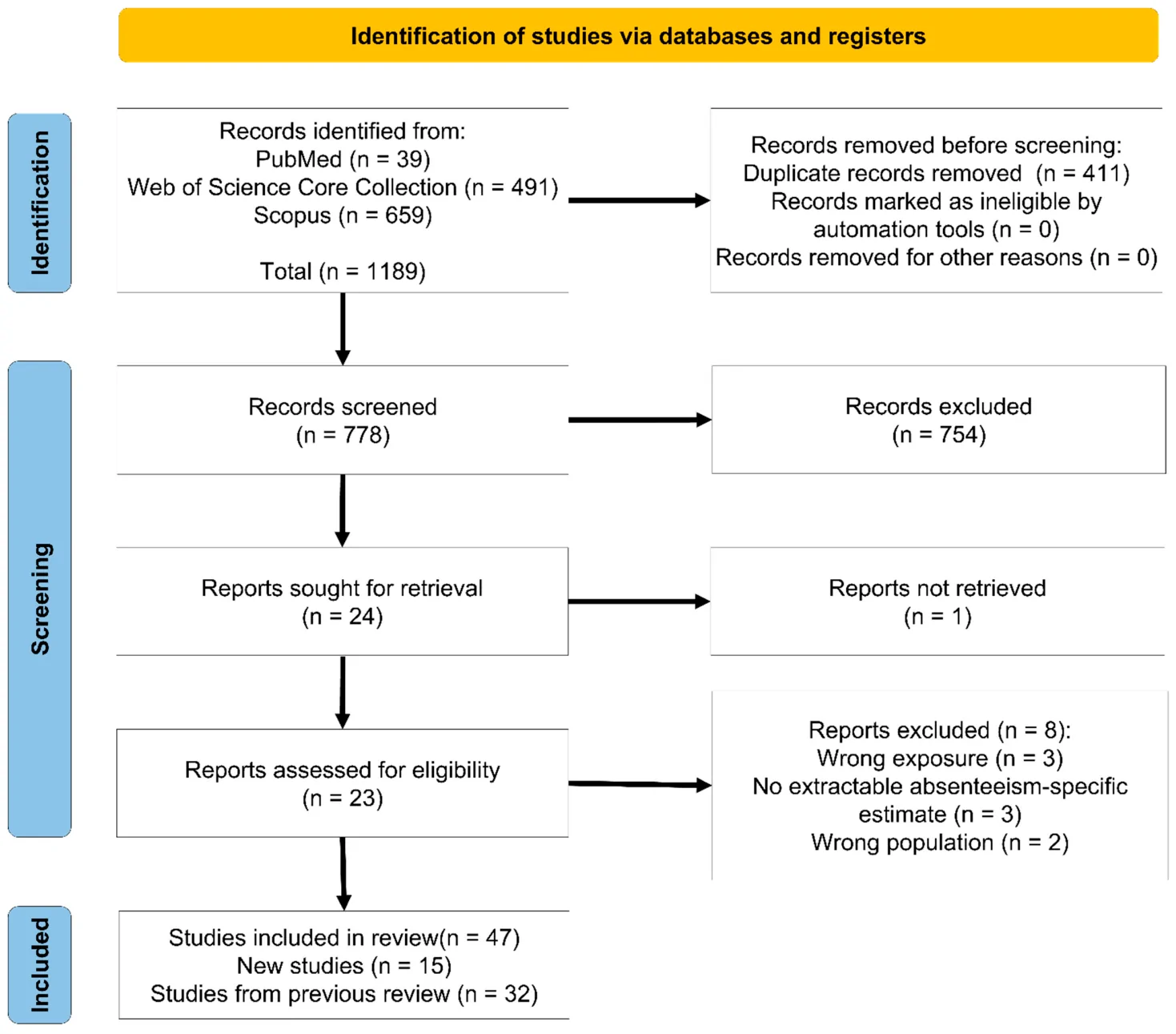
PRISMA 2020 flow diagram for study selection. The diagram shows the updated-search pathway from 7 June 2018 to 27 May 2026. The updated search identified 15 newly included studies. In addition, 32 studies from the previous systematic review were reassessed against the current eligibility criteria and retained. Therefore, the final review included 47 studies in total. Reports excluded after full-text assessment and specific reasons for exclusion are provided in Supplementary Table S9.

**Figure 2:**
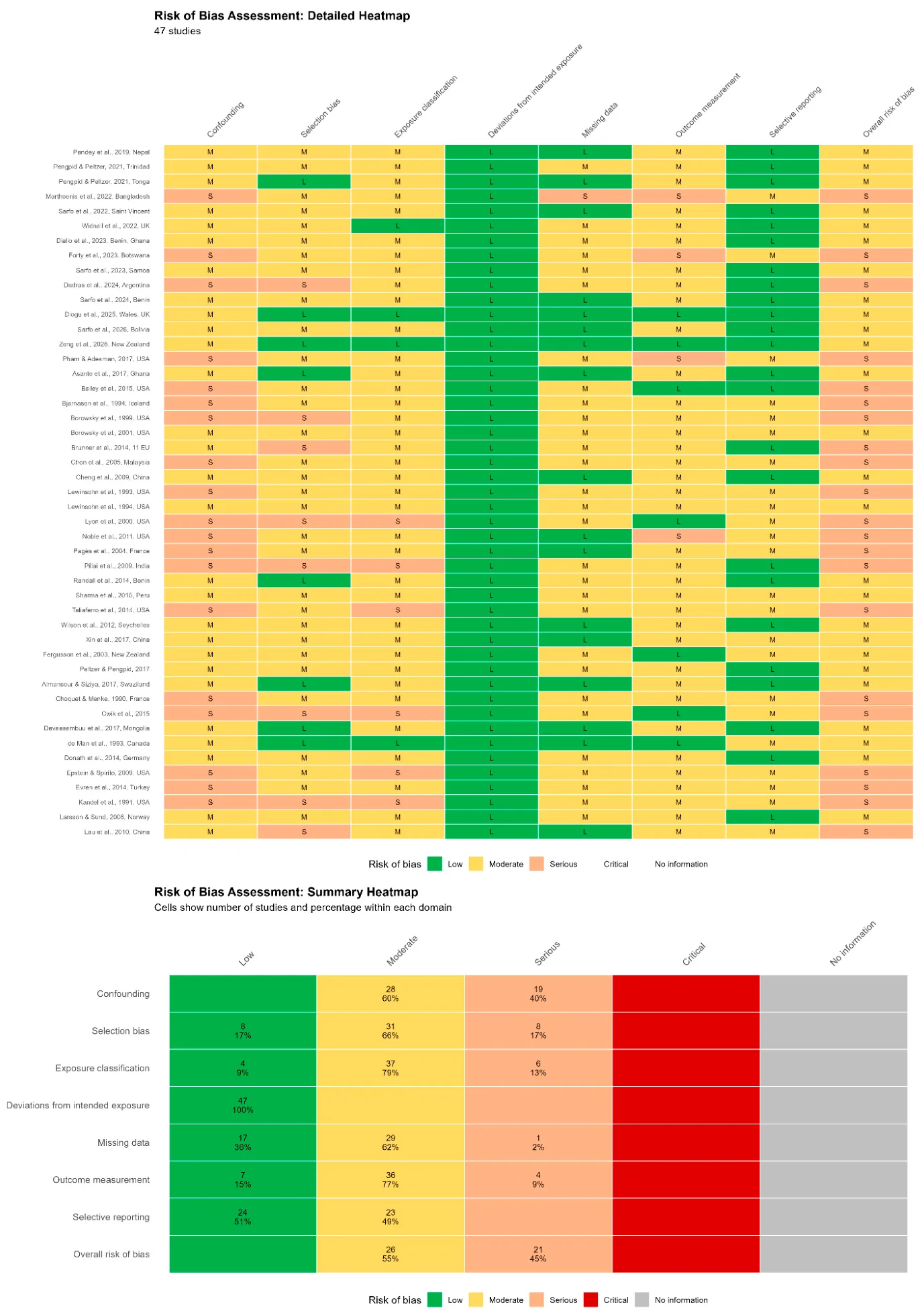
Risk of bias assessment of included studies using an adapted ROBINS-I framework. Overall risk of bias was moderate in 26 studies and serious in 21 studies. No study was rated as low or critical overall risk of bias. The main concerns related to confounding, selection bias, and exposure classification. The included studies shown in the figure are cited as [3,5,6,7,8,9,10,11,12,13,14,15,16,17,18,19,20,21,22,23,24,25,26,27,28,29,30,31,32,33,34,35,36,37,38,39,40,41,42,43,44,45,46,47,48,49,50].

**Figure 3:**
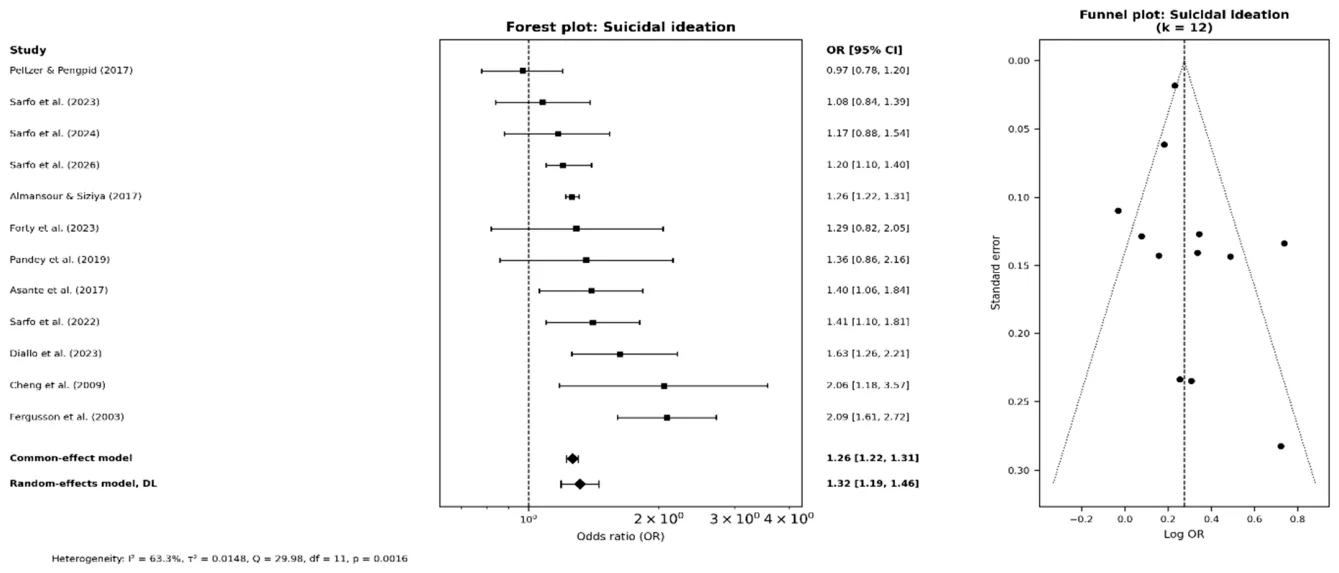
Forest and funnel plots of pooled odds ratios for suicidal ideation. Forest plot showing study-specific odds ratios (ORs) and 95% confidence intervals (CIs) for suicidal ideation across 12 included study estimates. Squares represent individual study estimates, horizontal lines indicate 95% CIs, and diamonds represent pooled estimates. The common-effect model showed a pooled OR of 1.26 (95% CI: 1.22–1.31), while the DerSimonian–Laird random-effects model showed a pooled OR of 1.32 (95% CI: 1.19–1.46), indicating increased odds of suicidal ideation. Heterogeneity was moderate to substantial (I^2^ = 63.3%, τ^2^ = 0.0148, Q = 29.98, df = 11, *p* = 0.0016). The funnel plot displays log OR against standard error to visually assess potential small-study effects or publication bias; the funnel plot includes the same 12 estimates as the forest plot and Supplementary Table S3. The study references shown in the plot are [10,15,19,23,24,25,40,44,45,46,48,49].

**Figure 4:**
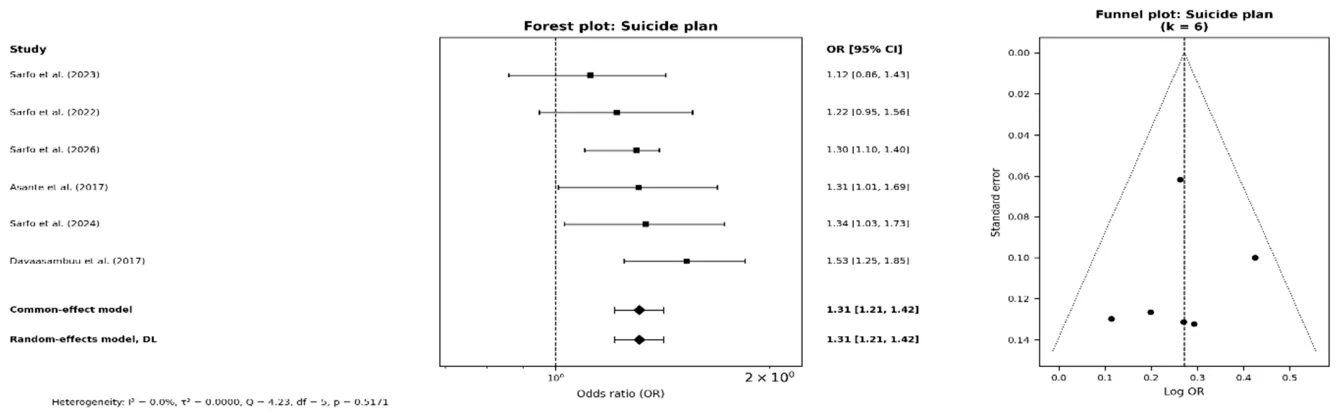
**Forest and funnel plots of pooled odds ratios for suicide plan. Forest plot showing study-specific odds ratios (ORs) and 95% confidence intervals (CIs) for suicide plan across six included study estimates.** Squares represent individual study estimates, horizontal lines indicate 95% CIs, and diamonds represent pooled estimates. Both the common-effect and DerSimonian–Laird random-effects models produced the same pooled estimate, with an OR of 1.31 (95% CI: 1.21–1.42), indicating increased odds of suicide plan. No meaningful heterogeneity was observed across studies (I^2^ = 0.0%, τ^2^ = 0.0000, Q = 4.23, df = 5, *p* = 0.5171). The funnel plot displays log OR against standard error to visually assess potential small-study effects or publication bias; the funnel plot includes the same six estimates as the forest plot and Supplementary Table S4. The study references shown in the plot are [25,28,44,46,48,49].

**Figure 5:**
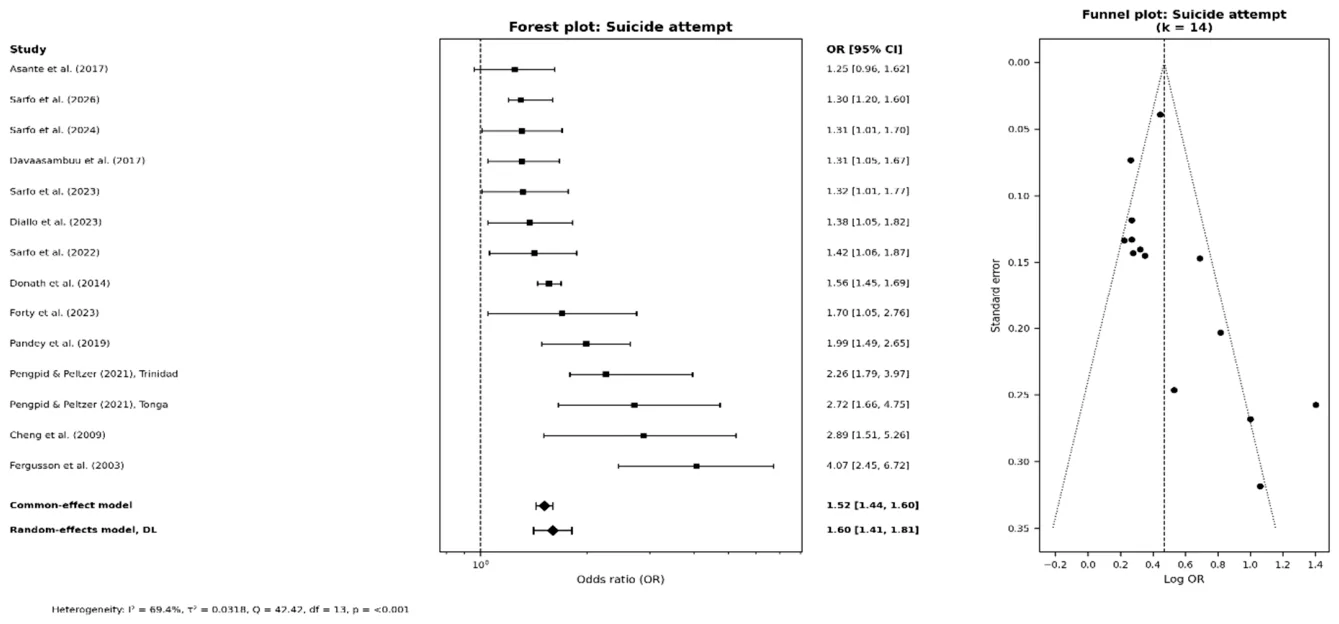
**Forest and funnel plots of pooled odds ratios for suicide attempt.** Forest plot showing study-specific odds ratios (ORs) and 95% confidence intervals (CIs) for suicide attempt across 14 included study estimates. Squares represent individual study estimates, horizontal lines indicate 95% CIs, and diamonds represent pooled estimates. The common-effect model showed a pooled OR of 1.52 (95% CI: 1.44–1.60), while the DerSimonian–Laird random-effects model showed a pooled OR of 1.60 (95% CI: 1.41–1.81), indicating increased odds of suicide attempt. Moderate to substantial heterogeneity was observed across studies (I^2^ = 69.4%, τ^2^ = 0.0318, Q = 42.42, df = 13, *p* < 0.001). The funnel plot displays log OR against standard error to visually assess potential small-study effects or publication bias; the funnel plot includes the same 14 estimates as the forest plot and Supplementary Table S5. The study references shown in the plot are [10,11,15,19,23,25,28,41,42,44,45,46,48,49].

**Table 1:** **Characteristics of included studies.**

References	Country	Data Collection	Study Design	N/Case-Controls	% Female	Age Range at Enrolment (Mean/Median)	Groups of Covariates
Almansour & Siziya, 2017 [24]	Swaziland	2013	Cross-sectional secondary analysis	*N* = 3680	51.2%	<11 to 18+ years	Age/gender; food security; psychosocial; friends; truancy; bullying/violence; substance use; parental understanding.
Oppong Asante et al., 2017 [25]	Ghana	2012	Cross-sectional secondary analysis	*n* = 1984	45.7%	≤14 to ≥18 years; majority ≥18 years	Age/sex; psychosocial; truancy; bullying/violence; food insecurity/friends; substance use; parental involvement/monitoring.
Bailey et al., 2015 [17]	USA	2006–2010	Retrospective record-linkage cohort/mortality study	168 injury-related deaths; 15 severe truancy history, 153 without; suicide deaths 2 vs. 27.	NR	11–17 years	None adjusted; severe truancy/contempt exposure; mortality person-years.
Bjarnason et al., 1994 [5]	Iceland	1992	Cross-sectional population-based school survey	N = 7018 valid questionnaires; 86.8% of national population in two cohorts.	50.0%	14–16 years	School, leisure, peer/friend, consumption and parent factors; suicide suggestion.
Borowsky et al., 1999 [6]	USA	1990	Cross-sectional school survey	*n* = 11,666	52.1%	12–18 years	Community/family/personal factors; suicide exposure; health/somatic/abuse; substance use; school/special ed.; gangs/guns; connectedness.
Borowsky et al., 2001 [7]	USA	1995–1996	Longitudinal cohort	n = 13,110	NR	Grades 7–12; exact age NR	Demographic, family and community factors; prior attempt; violence; substances; school/somatic problems; suicide exposure; treatment/weapons; sexuality; connectedness.
Brunner et al., 2014 [8]	EU	NR	Cross-sectional multicountry school survey	n = 12,068	55.7%	Mean 14.9 years (SD 0.89)	Demographics/country; religion/family context; parental support; social/victimisation; depression/anxiety/suicidality; risk behaviours.
Chen et al., 2005 [9]	Malaysia	2001	Cross-sectional school survey	N = 4500; 4454 answered suicidal ideation item.	53.6%	12–19 years; mean 15.3, median 15	Demographics; substances; family living; violence/injury/weapons; school safety/absence; sadness; drunk-driving exposure.
Cheng et al., 2009 [10]	China	2003	Cross-sectional school survey	N = 9015	51.0%	11–17 years	City/demographics; school/peer support; parental involvement/monitoring; missed classes.
Choquet & Menke, 1990 [26]	France	1988	Cross-sectional school survey	N = 1601; initial random sample 1722.	44.0%	13–16 years; mean approx. 14.7	None adjusted; health, drug use, delinquency, absence/lateness, violence and help-seeking.
Cwik et al., 2015 [27]	USA	NR	Cross-sectional community sample of adolescent suicide attempters	N = 71 suicide-attempting adolescents	64.8%	Mean 16.03 years (SD 1.8)	None adjusted; school status/safety absence; suicide/treatment/exposure; adverse events; family/community; depression/substance; cultural factors.
Davaasambuu et al., 2017 [28]	Mongolia	2013	Cross-sectional school survey	n = 5393	51.7%	12–17 years	Gender; psychosocial; substances; school fight/truancy; friends/peer support; bullying; residence.
De Man et al., 1993 [29]	Canada	NR	Cross-sectional school survey	N = 558; 272 males and 286 females.	51.3%	11–18 years; mean 14.0 (SD 1.46)	Sex/age; psychological traits; substances; stress/health; family/parenting; violence; academics/absenteeism; support/alienation.
Donath et al., 2014 [11]	Germany	2007–2008	Cross-sectional representative school survey	n = 44,610full model N around 44,134 depending on variables.	48.7%	Mean 15.3 years (SD 0.7)	Parenting; demographics; migration/welfare; substances/ADHD; family events; school grades/anxiety/integration/absenteeism; wellbeing/self-esteem.
Epstein & Spirito, 2009 [12]	USA	2005	Cross-sectional secondary analysis	N = 13,917 usable questionnaires	50.0%	12 to ≥18 years; grades 9–12	Substance use; aggression/victimisation; sexual risk; health; school safety/violence.
Evren et al., 2014 [30]	Turkey	2012	Cross-sectional online school survey	N = 4957; SHB n = 713, non-SHB n = 4244.	47.3%	Mean approx. 15.6 years	None for adjusted attendance exposure; substances; suicidality; mental health; impulsive/delinquent/aggressive/social factors; absenteeism measured.
Kandel et al., 1991 [31]	USA	1986	Cross-sectional school survey	N = 593 in methods; abstract reports 597.	NR overall	Exact age NR; 9th and 11th graders	None for attendance exposure; depressive symptoms, drugs/delinquency/eating, parent/peer/life events and school attachment.
Larsson & Sund, 2008 [32]	Norway	1998–1999	Prospective cohort school survey	T1 N = 2464; follow-up analytic n = 2360	50.8% at T1; 50.5% at follow-up	T1 mean 13.7 (range 12.5–15.7); T2 mean 14.9 (range 13.7–17.0)	Gender/age; adoption/puberty; somatic symptoms; stress/events; mental health/suicidality; suicide exposure; help-seeking.
Lau et al., 2010 [33]	China	2008	Cross-sectional school survey	N = 3324 secondary students	44.6%	Exact age NR; junior high grades 1–2 and senior high grades 1–2; age <15/≥15	Demographics/grade; prior adversity; earthquake impact; school absence; social support; media/disaster worry; PTSD/depression.
Lewinsohn et al., 1993 [34]	USA	1987–1989	Cross-sectional baseline analysis within community cohort	N = 1710 adolescents	52.9%	14–18 years; mean 16.6 (SD 1.2)	Gender; depression; internalising/externalising; past disorders; cognitions/coping; school/health problems; psychosocial risks.
Lewinsohn et al., 1994 [18]	USA	1987–1990	Prospective cohort	N = 1508 at follow-up; 26 future suicide attempts.	54.0%	14–18 years; mean 16.5 (SD 1.2)	Gender; depression; past attempt/ideation; friend attempt; self-esteem; support/coping; school absence/lateness/homework; health.
Lyon et al., 2000 [35]	USA	NR	Matched case–control clinical study	38 suicide attempters and 76 controls, matched 1:2 by age and gender.	82.2% cases; 81.6% controls	12–17 years; mean 14.7 cases, 14.9 controls	Insomnia; parent separation; substance abuse; neglect; academics/truancy/discipline; suicidal ideation; threats.
Noble et al., 2011 [3]	USA	NR	Matched case–control within cross-sectional school survey	Initial N = 7126; analytic matched N = 1276 (638 NSSI cases, 638 controls).	74.6% analytic sample	11–19 years; analytic mean 14.89 (SD 1.48)	School trust; unsafe absence; weapon/threat/bullying/fights at school.
Pagès et al., 2004 [36]	France	1999	Cross-sectional national school survey	Analysed N = 11,718; lifetime suicide attempters n = 1078; hospitalized n = 234.	51.9%	11–21 years; mean 16.6	Age; school/family/peer factors; self-esteem/depression; substances; delinquency/fighting/running away; absenteeism.
Pillai et al., 2009 [16]	India	2006	Cross-sectional community survey	Total N = 3662 youth; school/college variables available only for school/college-goer subgroup.	51.4%	16–24 years; mean 19.4 (SD 2.5)	Demographics/residence; school status/attendance; parent communication/independence; sex/abuse; mental disorder; substance use.
Randall et al., 2014 [13]	Benin	2009	Cross-sectional school survey; multinomial logistic regression	N = 2690 adolescents	Approx. 32.9%	Age categories ≤ 12, 13, 14, 15, ≥16; about 49.2% ≥ 16	Age/sex; psychosocial; bullying/violence; truancy; food insecurity; substances; parent/friend support.
Sharma et al., 2015 [37]	Peru	2014	Cross-sectional school survey	N = 916 secondary school adolescents	53.6%	12–18 years; median 15	Demographics; violence/family violence; absenteeism; parental support; health/happiness/body image; substances; sex/counselling.
Taliaferro et al., 2014 [14]	USA	2010	Cross-sectional population-based school survey	N = 70,722 by sex-specific methods counts (abstract reports 70,022).	50.0%	Exact age NR; grades 9 and 12	Demographics/SES/family; bullying/abuse/dating violence; health/substances; running away/self-injury; school violence/safety; weight/sexuality; mood/stress/impulsivity.
Wilson et al., 2012 [38]	Seychelles	NR	Cross-sectional GSHS secondary analysis	N = 1432 surveyed; analytic categories total 1427.	52.0%	11–17 years; mean 14.0 (SD 1.5)	Age/sex; deprivation; psychosocial; bullying/truancy; parental support/monitoring; friends; substances.
Xin et al., 2017 [39]	China	NR	Cross-sectional school-based survey	N = 11,880	50.5%	10–20 years; mean 14.62	Age; paternal education; risky behaviours; suicidality; impulsivity; depression/self-esteem/anxiety/affect; somatic symptoms.
Fergusson et al., 2003 [19]	New Zealand	1977–1998	Prospective longitudinal birth cohort	Original cohort N = 1265; analyses based on N = 1063 with suicidal-behaviour data.	49.8%	Enrolled at birth; outcomes measured approx. 15–21 years	Depression/family suicide/abuse; personality/self-esteem; school achievement/truancy/suspension; peers; individual/family/peer factors.
Peltzer & Pengpid, 2017 [40]	Cambodia	2007–2013	Cross-sectional pooled GSHS secondary analysis	N = 30,284 school children aged 13–15	51.5%	13–15 years; mean 14.1 (SD 0.8)	Demographics/country income; hunger/social isolation; bullying/violence; parent support; substances; activity/sedentary/BMI; truancy.
Pandey et al., 2019 [15]	Nepal	2015	Cross-sectional secondary analysis	N = 6531 participated; 6529 usable questionnaires.	NR	11–18 years; grades 7–11	Demographics; food insecurity/psychosocial; friends; truancy; bullying/violence; substances; parental involvement/privacy/support.
Pengpid & Peltzer, 2021 [41]	Trinidad	2017	Cross-sectional secondary analysis	N = 3869 school adolescents	51.8%	Median 14 years (IQR 3)	Sociodemographic/psychosocial; friends/peer support; bullying/violence; hunger; parental factors; truancy; substances/diet/sex/injuries.
Pengpid & Peltzer, 2021 [42]	Tonga	2017	Cross-sectional secondary analysis	N = 3333 school adolescents	49.3%	Median 14 years (IQR 3); age ≤13, 14–15, ≥16	Age/gender; psychosocial; friends/peer support; bullying/violence; hunger; parental factors; truancy; substances/diet/injuries.
Marthoenis et al., 2022 [43]	Bangladesh	2014–2015	Cross-sectional secondary analysis	N = 9052; Bangladesh 2570, Indonesia 6482.	58.4%	≤11 to ≥18 years; 48.7% aged 14–15	Age/gender; missed class; violence/bullying; psychosocial/hunger; diet; substances; sedentary behaviour; parental support/monitoring; friends.
Sarfo et al., 2022 [44]	Saint Vincent	2018	Cross-sectional secondary analysis	N = 1877	52.9%	Mainly 13–17 years; categories ≤15 and ≥16; grades 1–3 and 4–5	Demographics/grade; truancy; substances; violence/bullying/cyberbullying; parental factors; sex; injury; friends/psychosocial.
Widnall et al., 2022 [20]	UK	2009–2013	Retrospective cohort/linked administrative data study	N = 113,286 adolescents; 1020 ED self-harm presentations; 763 self-harm cases matched to NPD.	Approx. 50.1%	11–17 years	Demographics/SES; SEN/learning/behavioural needs; school type/attainment; poor attendance/exclusion; neurodevelopmental/mental health diagnoses.
Diallo et al., 2023 [23]	Benin	2012–2017	Cross-sectional pooled secondary analysis	N = 9726; Benin 2536; Ghana 1648; Liberia 2744; Sierra Leone 2798.	42.3%	Age ≤ 12, 13–15, ≥16; 48.5% aged ≥ 16	Demographics; food insecurity/sleep worry; social isolation; truancy; bullying/violence; cigarettes/drugs/intoxication; parental monitoring/privacy.
Forty et al., 2023 [45]	Botswana	2010	Cross-sectional secondary analysis	N = 3863 school-going adolescents	55.7%	10–19 years; age groups 10–12, 13–15, 16–19	Demographics/school/residence; self-confidence; missed class; hunger; substances; bullying/violence/injury; family/teacher emotional abuse.
Sarfo et al., 2023 [46]	Samoa	2017	Cross-sectional secondary analysis	N = 1779 school-going adolescents	63.0%	13–17 years; categories 13–15 and ≥16	Demographics/grade; truancy; hunger; substances; violence/injury/bullying; psychosocial/friends; parental tobacco/smoke; sex; parental monitoring/support.
Dadras et al., 2024 [47]	Argentina	2018	Cross-sectional secondary analysis; restricted to adolescents with suicidal thoughts	Full respondents N = 56,898; suicidal-ideator analytic sample N = 11,962; attempts among ideators n = 6033.	69.16% among suicidal ideators	Age < 14, 14–15, ≥16	Demographics/grade; psychosocial/hunger; bullying/violence; truancy/absenteeism; parental support; activity; substances; sexual risk.
Sarfo et al., 2024 [48]	Benin	2016	Cross-sectional secondary analysis	National dataset N = 2536; Table 1 analytic sample N = 2496.	45.7%	13–18 years; categories 13–15 and 16–18; grade categories 3rd–6th and terminal-2nd	Demographics/grade; truancy; hunger/sedentary/missed PE; substances; violence/injury/bullying; psychosocial/friends; sex; parental factors.
Diogu et al., 2025 [21]	Wales, UK	2012/13–2021	Retrospective electronic cohort/record-linkage study	N = 584,394 pupils; persistent absence n = 50,213; ever excluded n = 25,106; suicide deaths n = 123.	48.9%	Mean age at cohort entry 8.7 years; age < 10, 10–15, 16+	Demographics/SES; persistent absence/exclusion; self-harm; mental health, neurodevelopmental and learning disorders; alcohol/drug misuse.
Sarfo et al., 2026 [49]	Bolivia	2018	Cross-sectional secondary analysis	N = 7822 in-school adolescents	Approx. 48.5% (calculated from Table 1)	Mainly 13–18 years; 2nd Secondary-6th Secondary	Demographics; violence/injury; psychosocial/hunger/friends; alcohol/marijuana/inactivity/truancy; parental involvement.
Zeng et al., 2026 [22]	New Zealand	2015–2021	Retrospective longitudinal matched case–control/nested case–control study	Source cohort N = 562,455; attendance-linked cohort N = 544,455; 3042 cases and 12,168 matched controls.	75.8% in cases and controls (matched by sex)	School years 6–13; exact age NR	Student factors; school factors; previous suicidality; repeated/chronic/moderate absenteeism.
Pham & Adesman, 2017 [50]	USA	2015	Cross-sectional secondary analysis	N = 15,624 high-school students	51.3%	Grades 9–12; exact age NR	Sex/grade/race; bullying/cyberbullying; unsafe skipping/school violence; sadness sensitivity model.

**Table 2:** Summary of pooled findings and certainty of evidence.

Outcome	Studies/Estimates	Participants	Pooled Effect	Main Limitations	Certainty	Interpretation
Suicidal ideation	12 estimates	Not consistently extractable across all estimates	OR = 1.32, 95% CI: 1.19–1.46; I^2^ = 63.3%	Observational/cross-sectional evidence; no low-risk studies; heterogeneity; residual confounding and reverse causation possible	Low	Low-certainty evidence suggests an association
Suicide plan	6 estimates	Not consistently extractable across all estimates	OR = 1.31, 95% CI: 1.21–1.42; I^2^ = 0.0%	Observational evidence; no low-risk studies; variation in exposure definitions and adjustment sets	Low	Low-certainty evidence suggests an association
Suicide attempt	14 estimates	Not consistently extractable across all estimates	OR = 1.60, 95% CI: 1.41–1.81; I^2^ = 69.4%	Observational/cross-sectional evidence; no low-risk studies; heterogeneity; residual confounding and reverse causation possible	Low	Low-certainty evidence suggests an association

**Note:** Participant totals were not consistently extractable across all included estimates; therefore, the number of estimates is reported as the primary denominator for the quantitative synthesis. Domain-level certainty-of-evidence judgements are provided in Supplementary Table S10.

## Data Availability

The extracted data used in the review are provided in the Supplementary Material. Additional materials, including study-specific effect estimates, risk-of-bias assessments, dataset-overlap assessment, sensitivity analyses, and full-text exclusion details, are available as Supplementary Tables S3–S9.

## Supplementary Materials

The following supporting information can be downloaded at https://www.mdpi.com/article/10.3390/children13101373/s1: Supplementary File S1: PRISMA 2020 checklist; Supplementary File S2: Search strategy and effect estimates; Supplementary File S3: Risk-of-bias assessment table; Supplementary File S4: Dataset-overlap assessment, sensitivity analyses, and certainty-of-evidence judgements; Supplementary File S5: Excluded full-text reports; Supplementary File S6: Final analytical dataset; Supplementary File S7: R code to reproduce the analyses.
