# Supplementary material for: School Absenteeism and Self-Harm or Suicidal Outcomes Among Children, Adolescents, and School-Aged Young People: A Systematic Review and Meta-Analysis
Source: Children (Basel). 2026 Oct 10;13(10):1373. doi: 10.3390/children13101373 (PMC13703334; doi:10.3390/children13101373)
Supplement: Supplementary file 1 [file children-13-01373-s001.zip › Supplementary File S5. Excluded full-text reports.pdf]

Supplementary Table S9. Full-text reports excluded after eligibility assessment

This table lists the eight reports excluded after full-text assessment in the updated search pathway and gives the specific reason for exclusion. Reasons correspond to the categories shown in the PRISMA flow diagram and Results section.

No.	Report citation	Reason for exclusion	Specific explanation
1	Williams J III, Richardson S, Lewis CW, Alexander J. Investigating disciplinary risk in urban high schools as a precursor for suicide-related behaviors among Black students. J Trauma Stud Educ. 2022;1(3):90-107. doi:10.32674/jtse.v1i3.5037.	Wrong exposure	The study examined school disciplinary risk, particularly out-of-school suspension, rather than school absenteeism, truancy, unauthorised absence, school refusal, or chronic/persistent attendance problems as the exposure of interest.
2	Peprah P, Yeboah-Asiamah Asare B, Okwei R, Agyemang-Duah W, Osafo J, Kretchy IA, Gyasi RM. A moderated mediation analysis of the association between smoking and suicide attempts among adolescents in 28 countries. Sci Rep. 2023;13:5755. doi:10.1038/s41598-023-32610-8.	No extractable absenteeism-specific estimate	Truancy was analysed as a mediator in the association between cigarette smoking and suicide attempt, not as the main attendance-related exposure. No direct absenteeism/truancy-specific effect estimate suitable for the quantitative synthesis was reported.
3	Szmajda R, Mokros L, Szmajda-Krygier D, Gmitrowicz A. Factors associated with suicide attempt risk in adolescent inpatient psychiatric care: toward a practical model. Eur Child Adolesc Psychiatry. 2024;33:1875-1881. doi:10.1007/s00787-023-02272-y.	Wrong population	The sample consisted of adolescent psychiatric inpatients rather than a school-enrolled or community-based adolescent population. Although truancy was reported as one psychosocial factor, the inpatient setting did not match the target population for the updated quantitative synthesis.
4	Potard C, Combes C, Labrell F. Suicidal ideation among French adolescents: separation anxiety and attachment according to sex. J Genet Psychol. 2020;181(6):470-488. doi:10.1080/00221325.2020.1822773.	Wrong exposure	The study focused on parental attachment, separation anxiety and school phobia in relation to suicidal ideation. It did not measure school absenteeism or a comparable school attendance exposure for the review.

Supplementary Table S9. Full-text reports excluded after eligibility assessment (continued)

No.	Report citation	Reason for exclusion	Specific explanation
5	Fadum EA, Carlsen EO, Ramberg M, Strand LA, Haberg SE, Borud E, Martinussen M. Social and life skills in adolescents who have self-harmed: analysis of survey responses from a national sample of adolescents in Norway. BMJ Open. 2021;11:e054707. doi:10.1136/bmjopen-2021-054707.	Wrong exposure	The attendance-related item concerned school/work absence due to injury or illness over an eight-week period. This was not an eligible attendance-related construct such as truancy, unauthorised absence, school refusal/avoidance, or chronic/persistent poor attendance.
6	Hasan MM, Fatima Y, Pandey S, Tariqujjaman M, Cleary A, Baxter J, Mamun AA. Pathways linking bullying victimisation and suicidal behaviours among adolescents. Psychiatry Res. 2021;302:113992. doi:10.1016/j.psychres.2021.113992.	No extractable absenteeism-specific estimate	The main exposure was bullying victimisation. Truancy was reported as a characteristic or pathway-related variable, but the report did not provide a direct attendance-related exposure estimate for suicidal ideation, suicide plan or suicide attempt.
7	McEvoy D, Brannigan R, Walsh C, Arensman E, Clarke M. Identifying high-risk subgroups for self-harm in adolescents and young adults: a longitudinal latent class analysis of risk factors. J Affect Disord. 2024;351:40-48. doi:10.1016/j.jad.2024.01.230.	No extractable absenteeism-specific estimate	Truancy or excessive absence from school was included within a broader latent class/risk-profile variable. The independent effect of absenteeism could not be separated from other co-occurring risk factors, so no absenteeism-specific estimate was extractable.
8	Komura Y, Ema S, Nomura N, Kodama K, Takabatake S, Misawa F, Takeuchi H. Parenting style and deliberate self-harm among children and adolescents: a hospital-based case-control study in Japan. J Psychiatr Res. 2026;195:317-323. doi:10.1016/j.jpsychires.2026.01.025.	Wrong population	The study used a hospital-based psychiatric case-control sample and the primary exposure was parenting style. School refusal was included only as an adjustment covariate, not as an attendance-related exposure of interest.
