# Supplementary material for: School Absenteeism and Self-Harm or Suicidal Outcomes Among Children, Adolescents, and School-Aged Young People: A Systematic Review and Meta-Analysis
Source: Children (Basel). 2026 Oct 10;13(10):1373. doi: 10.3390/children13101373 (PMC13703334; doi:10.3390/children13101373)
Supplement: Supplementary file 1 [file children-13-01373-s001.zip › Supplementary File S1. PRISMA 2020 checklist.pdf]

Supplementary File S1. PRISMA 2020 checklist

Manuscript: School Absenteeism and Self-Harm or Suicidal Outcomes among Children, Adolescents, and School-Aged Young People: A Systematic Review and Meta-Analysis

Manuscript ID: children-4534249

**Section**	**Item**	**Topic**	**Checklist item**	**Location in manuscript**
TITLE	1	Title	Identify the report as a systematic review.	Title page
ABSTRACT	2	Abstract	See the PRISMA 2020 for Abstracts checklist.	Abstract
INTRODUCTION	3	Rationale	Describe the rationale for the review in the context of existing knowledge.	Introduction, paragraphs 1–6
	4	Objectives	Provide an explicit statement of the objective(s) or question(s) the review addresses.	Introduction, final paragraph
METHODS	5	Eligibility criteria	Specify inclusion and exclusion criteria for the review and how studies were grouped for syntheses.	Methods 2.2
	6	Information sources	Specify all databases, registers, websites, organisations, reference lists and other sources searched or consulted, and the date when each source was last searched.	Methods 2.3–2.4; Supplementary Table S1
	7	Search strategy	Present the full search strategies for all databases, registers and websites, including any filters and limits used.	Methods 2.4; Supplementary Table S1
	8	Selection process	Specify methods used to decide whether a study met inclusion criteria, including number of reviewers and automation tools if used.	Methods 2.5
	9	Data collection process	Specify methods used to collect data from reports, including number of reviewers and processes for obtaining or confirming data.	Methods 2.6
	10a	Data items: outcomes	List and define all	Methods 2.2 and 2.6

			outcomes for which data were sought and whether all results compatible with each outcome were sought.	
	10b	Data items: other variables	List and define all other variables for which data were sought.	Methods 2.6; Table 1
	11	Study risk of bias assessment	Specify methods used to assess risk of bias in included studies.	Methods 2.7; Results 3.3; Supplementary Table S6
	12	Effect measures	Specify for each outcome the effect measures used in the synthesis or presentation of results.	Methods 2.8
	13a	Synthesis methods: eligibility for synthesis	Describe processes used to decide which studies were eligible for each synthesis.	Methods 2.8; Supplementary Tables S3–S5
	13b	Synthesis methods: data preparation	Describe methods required to prepare data for presentation or synthesis.	Methods 2.6–2.8
	13c	Synthesis methods: tabulation/visual display	Describe methods used to tabulate or visually display results of individual studies and syntheses.	Methods 2.8; Figures 3–5; Supplementary Tables S3–S5
	13d	Synthesis methods: meta-analysis model	Describe methods used to synthesize results and rationale for models.	Methods 2.8; Supplementary Table S8
	13e	Synthesis methods: heterogeneity	Describe methods used to explore possible causes of heterogeneity.	Methods 2.8; Results 3.8–3.9; Supplementary Table S8
	13f	Synthesis methods: sensitivity analyses	Describe sensitivity analyses conducted to assess robustness.	Methods 2.8; Results 3.9; Supplementary Table S8
	14	Reporting bias assessment	Describe methods used to assess risk of bias due to missing results.	Methods 2.8; Results 3.4–3.6; Figures 3–5
	15	Certainty assessment	Describe methods used to assess certainty or confidence in the body of evidence.	Methods 2.9; Results 3.10; Table 2
RESULTS	16a	Study selection: search results	Describe results of search and selection process, from number of records identified to studies included.	Results 3.1; Figure 1
	16b	Study selection: excluded studies	Cite studies that appeared to meet inclusion criteria but were excluded, and	Results 3.1; Figure 1; Supplementary Table S9 (full-text reports excluded after

			explain why.	eligibility assessment, with citations and specific reasons)
	17	Study characteristics	Cite each included study and present its characteristics.	Results 3.2; Table 1
	18	Risk of bias in studies	Present assessments of risk of bias for each included study.	Results 3.3; Figure 2; Supplementary Table S6
	19	Results of individual studies	For all outcomes, present summary statistics and effect estimates for each study.	Supplementary Tables S3–S5; Figures 3–5
	20a	Results of syntheses: characteristics and risk of bias	For each synthesis, briefly summarise characteristics and risk of bias among contributing studies.	Results 3.4–3.6; Results 3.10; Supplementary Tables S3–S6
	20b	Results of syntheses: statistical results	Present results of all statistical syntheses.	Results 3.4–3.6; Figures 3–5; Supplementary Table S8
	20c	Results of syntheses: heterogeneity	Present results of investigations of possible causes of heterogeneity.	Results 3.8–3.9; Supplementary Table S8
	20d	Results of syntheses: sensitivity analyses	Present results of sensitivity analyses.	Results 3.9; Supplementary Table S8
	21	Reporting biases	Present assessments of risk of bias due to missing results.	Results 3.4–3.6; Figures 3–5
	22	Certainty of evidence	Present assessments of certainty or confidence in the body of evidence.	Results 3.10; Table 2
DISCUSSION	23a	Discussion: general interpretation	Provide a general interpretation of results in the context of other evidence.	Discussion 4.1–4.3
	23b	Discussion: limitations of evidence	Discuss limitations of evidence included in the review.	Discussion 4.5
	23c	Discussion: limitations of review process	Discuss limitations of review processes used.	Discussion 4.5
	23d	Discussion: implications	Discuss implications of results for practice, policy, and research.	Discussion 4.4–4.6
OTHER INFORMATION	24a	Registration and protocol	Provide registration information, including register name and registration number.	Methods 2.1; PROSPERO CRD420261472681
	24b	Protocol availability	Indicate where the review protocol can be accessed or state not publicly available.	Methods 2.1
	24c	Protocol amendments	Describe and explain	Methods 2.1; revised

			any amendments to information provided at registration or in the protocol.	search and sensitivity details in Methods 2.4 and 2.8
	25	Support	Describe sources of financial or non-financial support for the review.	Declarations: Funding
	26	Competing interests	Declare competing interests of review authors.	Declarations: Competing interests
	27	Availability of data, code and materials	Report which data, code and materials are publicly available and where they can be found.	Declarations: Data availability; Supplementary Files 1–4
