# Supplementary material for: School Absenteeism and Self-Harm or Suicidal Outcomes Among Children, Adolescents, and School-Aged Young People: A Systematic Review and Meta-Analysis
Source: Children (Basel). 2026 Oct 10;13(10):1373. doi: 10.3390/children13101373 (PMC13703334; doi:10.3390/children13101373)
Supplement: Supplementary file 1 [file children-13-01373-s001.zip › Supplementary File S2. Search strategy and effect estimates.pdf]

Supplementary File S2. Search strategy and effect estimates

Definitive analytical source. The study-specific estimates listed in Supplementary Tables S3–S5 are the final analytical dataset used to generate Figures 3–5, the corresponding funnel plots, heterogeneity statistics, pooled estimates, and sensitivity analyses. The machine-readable dataset and reproducibility code are provided as Supplementary Files S6 and S7.

Supplementary Table S1. Database-specific search strategy for PubMed, Scopus, and Web of Science

Concept	PubMed	Scopus Advanced	Web of Science Core Collection
Population	("Child"[Mesh] OR "Adolescent"[Mesh] OR "Students"[Mesh] OR child*[tiab] OR adolescen*[tiab] OR student*[tiab] OR youth*[tiab] OR pupil*[tiab] OR schoolchild*[tiab] OR teen*[tiab] OR pediatric*[tiab] OR paediatric*[tiab] OR "young people"[tiab] OR "young person"[tiab])	TITLE-ABS-KEY(child* OR adolescen* OR student* OR youth* OR pupil* OR schoolchild* OR teen* OR pediatric* OR paediatric* OR "young people" OR "young person")	TS=(child* OR adolescen* OR student* OR youth* OR pupil* OR schoolchild* OR teen* OR pediatric* OR paediatric* OR "young people" OR "young person")
Outcome	("Suicide"[Mesh] OR "Suicide, Attempted"[Mesh] OR "Suicidal Ideation"[Mesh] OR "Drug Overdose"[Mesh] OR "Self-Injurious Behavior"[Mesh] OR suicid*[tiab] OR "suicidal ideation"[tiab] OR "suicide attempt"[tiab] OR "suicide attempts"[tiab] OR "suicidal behavior"[tiab] OR "suicidal behaviour"[tiab] OR overdose*[tiab] OR NSSI[tiab] OR "non-suicidal self-injury"[tiab] OR "nonsuicidal self-injury"[tiab] OR "non suicidal self injury"[tiab] OR autoaggress*[tiab] OR automutilat*[tiab] OR cutt*[tiab] OR selfharm*[tiab] OR "self harm"[tiab] OR "self-harm"[tiab] OR "self harming"[tiab] OR "self-harming"[tiab] OR selfinjur*[tiab] OR "self injury"[tiab] OR "self-injury"[tiab] OR "self injurious"[tiab] OR "self-injurious"[tiab] OR selfmutilat*[tiab] OR "self mutilation"[tiab] OR "self-mutilation"[tiab] OR selfpoison*[tiab] OR "self poisoning"[tiab] OR "self-poisoning"[tiab] OR selfdestruct*[tiab] OR "self destructive"[tiab] OR "self-destructive"[tiab] OR selfinflict*[tiab] OR "self inflicted"[tiab] OR "self-inflicted"[tiab] OR selfimmolat*[tiab] OR "self immolation"[tiab] OR "self-immolation"[tiab])	TITLE-ABS-KEY(suicid* OR "suicidal ideation" OR "suicide attempt" OR "suicide attempts" OR "suicidal behavior" OR "suicidal behaviour" OR overdose* OR NSSI OR "non-suicidal self-injury" OR "nonsuicidal self-injury" OR "non suicidal self injury" OR autoaggress* OR automutilat* OR cutt* OR selfharm* OR "self harm" OR "self-harm" OR "self harming" OR "self-harming" OR selfinjur* OR "self injury" OR "self-injury" OR "self injurious" OR "self-injurious" OR selfmutilat* OR "self mutilation" OR "self-mutilation" OR selfpoison* OR "self poisoning" OR "self-poisoning" OR selfdestruct* OR "self destructive" OR "self-destructive" OR selfinflict* OR "self inflicted" OR "self-inflicted" OR selfimmolat* OR "self immolation" OR "self-immolation" OR (self W/2 cut*) OR (self W/2 harm*) OR (self W/2 injur*) OR (self W/2 mutilat*) OR (self W/2 poison*) OR (self W/2 destruct*) OR (self W/2 inflict*) OR (self W/2 immolat*))	TS=(suicid* OR "suicidal ideation" OR "suicide attempt" OR "suicide attempts" OR "suicidal behavior" OR "suicidal behaviour" OR overdose* OR NSSI OR "non-suicidal self-injury" OR "nonsuicidal self-injury" OR "non suicidal self injury" OR autoaggress* OR automutilat* OR cutt* OR selfharm* OR "self harm" OR "self-harm" OR "self harming" OR "self-harming" OR selfinjur* OR "self injury" OR "self-injury" OR "self injurious" OR "self-injurious" OR selfmutilat* OR "self mutilation" OR "self-mutilation" OR selfpoison* OR "self poisoning" OR "self-poisoning" OR selfdestruct* OR "self destructive" OR "self-destructive" OR selfinflict* OR "self inflicted" OR "self-inflicted" OR selfimmolat* OR "self immolation" OR "self-immolation" OR (self NEAR/2 cut*) OR (self NEAR/2 harm*) OR (self NEAR/2 injur*) OR (self NEAR/2 mutilat*) OR (self NEAR/2 poison*) OR (self NEAR/2 destruct*) OR (self NEAR/2 inflict*) OR (self NEAR/2 immolat*))
Exposure	("Absenteeism"[Mesh] OR absenteeism[tiab] OR truan*[tiab] OR truancy[tiab] OR "school attendance"[tiab] OR "poor attendance"[tiab] OR "poor school attendance"[tiab] OR "school absenteeism"[tiab] OR "school absence"[tiab] OR "school absences"[tiab] OR "school refusal"[tiab] OR "school phobia"[tiab] OR "school nonattendance"[tiab] OR "school non-attendance"[tiab] OR "school non attendance"[tiab] OR "chronic	TITLE-ABS-KEY(absenteeism OR truan* OR truancy OR "school attendance" OR "poor attendance" OR "poor school attendance" OR "school absenteeism" OR "school absence" OR "school absences" OR "school refusal" OR "school phobia" OR "school nonattendance" OR "school non-attendance" OR "school non attendance" OR "chronic absenteeism" OR "persistent absence" OR "persistent absenteeism" OR "unauthorised absence" OR "unauthorized absence"	TS=(absenteeism OR truan* OR truancy OR "school attendance" OR "poor attendance" OR "poor school attendance" OR "school absenteeism" OR "school absence" OR "school absences" OR "school refusal" OR "school phobia" OR "school nonattendance" OR "school non-attendance" OR "school non attendance" OR "chronic absenteeism" OR "persistent absence" OR "persistent absenteeism" OR "unauthorised absence" OR "unauthorized absence"

Concept	PubMed	Scopus Advanced	Web of Science Core Collection
	absenteeism"[tiab] OR "persistent absence"[tiab] OR "persistent absenteeism"[tiab] OR "unauthorised absence"[tiab] OR "unauthorized absence"[tiab] OR "missed school"[tiab] OR "miss school"[tiab] OR "missing school"[tiab] OR "skipped school"[tiab] OR "skipping school"[tiab] OR "skip school"[tiab] OR "missed class"[tiab] OR "missed classes"[tiab] OR "missing class"[tiab] OR "missing classes"[tiab] OR "skipped class"[tiab] OR "skipped classes"[tiab] OR "skipping class"[tiab] OR "skipping classes"[tiab] OR "skip class"[tiab] OR "skip classes"[tiab] OR ((school*[tiab] OR kindergarten[tiab] OR nursery[tiab] OR education*[tiab]) AND (attend*[tiab] OR nonattend*[tiab] OR refus*[tiab] OR absen*[tiab] OR phobi*[tiab] OR truan*[tiab])) OR (school*[tiab] AND (suspension[tiab] OR suspensions[tiab] OR suspen*[tiab] OR expulsion[tiab] OR expulsions[tiab] OR expel*[tiab] OR exclusion[tiab] OR exclusions[tiab] OR exclude*[tiab])) OR "school suspension"[tiab] OR "school suspensions"[tiab] OR "school expulsion"[tiab] OR "school expulsions"[tiab] OR "school exclusion"[tiab] OR "school exclusions"[tiab] OR "suspended from school"[tiab] OR "expelled from school"[tiab] OR "excluded from school"[tiab] OR "managed move"[tiab] OR "managed moves"[tiab] OR ((school*[tiab] OR educat*[tiab]) AND ("failure to complete"[tiab] OR terminat*[tiab] OR dropout*[tiab] OR "drop out"[tiab] OR "drop-out"[tiab])))	OR "missed school" OR "miss school" OR "missing school" OR "skipped school" OR "skipping school" OR "skip school" OR "missed class" OR "missed classes" OR "missing class" OR "missing classes" OR "skipped class" OR "skipped classes" OR "skipping class" OR "skipping classes" OR "skip class" OR "skip classes" OR (school* W/4 attend*) OR (school* W/4 absen*) OR (school* W/4 truan*) OR (school* W/4 refus*) OR (school* W/4 phobi*) OR (school* W/4 nonattend*) OR (school* W/4 non-attend*) OR (kindergarten W/4 attend*) OR (kindergarten W/4 absen*) OR (kindergarten W/4 refus*) OR (nursery W/4 attend*) OR (nursery W/4 absen*) OR (nursery W/4 refus*) OR (education* W/4 attend*) OR (education* W/4 absen*) OR (education* W/4 refus*) OR (education* W/4 truan*) OR "school suspension" OR "school suspensions" OR "school expulsion" OR "school expulsions" OR "school exclusion" OR "school exclusions" OR "suspended from school" OR "expelled from school" OR "excluded from school" OR "managed move" OR "managed moves" OR (school* W/5 suspen*) OR (school* W/5 expel*) OR (school* W/5 expulsion) OR (school* W/5 exclusion) OR (school* W/5 exclude*) OR (manage* W/1 move*) OR (terminat* W/5 school*) OR ("failure to complete" W/5 school*) OR ("failure to complete" W/5 educat*) OR (school* W/4 dropout*) OR (school* W/4 drop-out*))	OR "missed school" OR "miss school" OR "missing school" OR "skipped school" OR "skipping school" OR "skip school" OR "missed class" OR "missed classes" OR "missing class" OR "missing classes" OR "skipped class" OR "skipped classes" OR "skipping class" OR "skipping classes" OR "skip class" OR "skip classes" OR ((school* OR kindergarten OR nursery OR education*) NEAR/4 (attend* OR absen* OR truan* OR refus* OR phobi* OR nonattend* OR "non-attend*")) OR "school suspension" OR "school suspensions" OR "school expulsion" OR "school expulsions" OR "school exclusion" OR "school exclusions" OR "suspended from school" OR "expelled from school" OR "excluded from school" OR "managed move" OR "managed moves" OR (school* NEAR/5 suspen*) OR (school* NEAR/5 expel*) OR (school* NEAR/5 expulsion) OR (school* NEAR/5 exclusion) OR (school* NEAR/5 exclude*) OR (manage* NEAR/1 move*) OR (terminat* NEAR/5 school*) OR ("failure to complete" NEAR/5 school*) OR ("failure to complete" NEAR/5 educat*) OR (school* NEAR/4 dropout*) OR (school* NEAR/4 "drop-out*"))
Final search	#1 AND #2 AND #3 AND 2018/06/07:2026/05/27[dp]	#1 AND #2 AND #3 AND PUBYEAR > 2017 AND PUBYEAR < 2027	#1 AND #2 AND #3 AND DOP=(2018-06-07/2026-05-27)
Database notes	Use PubMed main search box. Date tag [dp] = publication date.	Use Advanced document search with Advanced query ON. Do not paste this into Basic Search or Scopus will wrap TITLE-ABS-KEY() again.	Use Web of Science Core Collection Advanced Search. If DOP is not accepted in your interface, search with PY=2018-2026 and manually exclude records before 7 June 2018.

Note. MeSH = Medical Subject Headings; tiab = title/abstract; TITLE-ABS-KEY = Scopus title, abstract and keyword fields; TS = Web of Science Topic field; DOP = Date of Publication.

Supplementary Table S2. Outcome-specific quantitative synthesis structure

Outcome domain	Quantitative synthesis status	Location of study-specific estimates
Suicidal ideation	Pooled meta-analysis conducted	Supplementary Table S3
Suicide plan	Pooled meta-analysis conducted	Supplementary Table S4
Suicide attempt	Pooled meta-analysis conducted	Supplementary Table S5
Non-suicidal self-injury, deliberate self-harm, composite suicidal behaviour, and suicide death	Narrative synthesis only because outcome definitions, exposure definitions, effect measures, or data sources were too heterogeneous for pooling	Main manuscript, Section 3.7

Supplementary Table S3. Study-specific estimates included in the suicidal ideation meta-analysis

Study	Outcome	OR	95% CI	log OR	SE(log OR)	Exposure category	Adjustment status
Peltzer & Pengpid (2017)	Suicidal ideation	0.97	0.78–1.20	-0.030459	0.109894	Truancy / skipped school / unauthorised absence	Adjusted
Sarfo et al. (2023)	Suicidal ideation	1.08	0.84–1.39	0.076961	0.128484	Truancy / skipped school / unauthorised absence	Adjusted
Sarfo et al. (2024)	Suicidal ideation	1.17	0.88–1.54	0.157004	0.142759	Truancy / skipped school / unauthorised absence	Adjusted
Sarfo et al. (2026)	Suicidal ideation	1.20	1.10–1.40	0.182322	0.061521	Truancy / skipped school / unauthorised absence	Adjusted
Almansour & Siziya (2017)	Suicidal ideation	1.26	1.22–1.31	0.231112	0.018157	Truancy / skipped school / unauthorised absence	Adjusted
Forty et al. (2023)	Suicidal ideation	1.29	0.82–2.05	0.254642	0.233748	Truancy / skipped school / unauthorised absence	Adjusted
Pandey et al. (2019)	Suicidal ideation	1.36	0.86–2.16	0.307485	0.234931	Truancy / skipped school / unauthorised absence	Adjusted
Oppong Asante et al. (2017)	Suicidal ideation	1.40	1.06–1.84	0.336472	0.140688	Truancy / skipped school / unauthorised absence	Adjusted
Sarfo et al. (2022)	Suicidal ideation	1.41	1.10–1.81	0.343590	0.127045	Truancy / skipped school / unauthorised absence	Adjusted
Diallo et al. (2023)	Suicidal ideation	1.63	1.26–2.21	0.488580	0.143337	Truancy / skipped school / unauthorised absence	Adjusted
Cheng et al. (2009)	Suicidal ideation	2.06	1.18–3.57	0.722706	0.282411	Truancy / skipped school / unauthorised absence	Adjusted
Fergusson et al. (2003)	Suicidal ideation	2.09	1.61–2.72	0.737164	0.133775	Truancy / skipped school / unauthorised absence	Adjusted

Supplementary Table S4. Study-specific estimates included in the suicide plan meta-analysis

Study	Outcome	OR	95% CI	log OR	SE(log OR)	Exposure category	Adjustment status
Sarfo et al. (2023)	Suicide plan	1.12	0.86–1.43	0.113329	0.129719	Truancy / skipped school / unauthorised absence	Adjusted
Sarfo et al. (2022)	Suicide plan	1.22	0.95–1.56	0.198851	0.126525	Truancy / skipped school / unauthorised absence	Adjusted
Sarfo et al. (2026)	Suicide plan	1.30	1.10–1.40	0.262364	0.061521	Truancy / skipped school / unauthorised absence	Adjusted
Oppong Asante et al. (2017)	Suicide plan	1.31	1.01–1.69	0.270027	0.131321	Truancy / skipped school / unauthorised absence	Adjusted
Sarfo et al. (2024)	Suicide plan	1.34	1.03–1.73	0.292670	0.132286	Truancy / skipped school / unauthorised absence	Adjusted
Davaasambuu et al. (2017)	Suicide plan	1.53	1.25–1.85	0.425268	0.100011	Truancy / skipped school / unauthorised absence	Adjusted

Supplementary Table S5. Study-specific estimates included in the suicide attempt meta-analysis

Study	Outcome	OR	95% CI	log OR	SE(log OR)	Exposure category	Adjustment status
Oppong Asante et al. (2017)	Suicide attempt	1.25	0.96–1.62	0.223144	0.133482	Truancy / skipped school / unauthorised absence	Adjusted
Sarfo et al. (2026)	Suicide attempt	1.30	1.20–1.60	0.262364	0.073388	Truancy / skipped school / unauthorised absence	Adjusted
Sarfo et al. (2024)	Suicide attempt	1.31	1.01–1.70	0.270027	0.132826	Truancy / skipped school / unauthorised absence	Adjusted
Davaasambuu et al. (2017)	Suicide attempt	1.31	1.05–1.67	0.270027	0.118376	Truancy / skipped school / unauthorised absence	Adjusted
Sarfo et al. (2023)	Suicide attempt	1.32	1.01–1.77	0.277632	0.143120	Truancy / skipped school / unauthorised absence	Adjusted
Diallo et al. (2023)	Suicide attempt	1.38	1.05–1.82	0.322083	0.140318	Truancy / skipped school / unauthorised absence	Adjusted
Sarfo et al. (2022)	Suicide attempt	1.42	1.06–1.87	0.350657	0.144814	Truancy / skipped school / unauthorised absence	Adjusted
Donath et al. (2014)	Suicide attempt	1.56	1.45–1.69	0.444686	0.039073	Other attendance-related measure	Adjusted
Forty et al. (2023)	Suicide attempt	1.70	1.05–2.76	0.530628	0.246541	Truancy / skipped school / unauthorised absence	Adjusted
Pandey et al. (2019)	Suicide attempt	1.99	1.49–2.65	0.688135	0.146884	Truancy / skipped school / unauthorised absence	Adjusted

Study	Outcome	OR	95% CI	log OR	SE(log OR)	Exposure category	Adjustment status
Pengpid & Peltzer (2021), Trinidad	Suicide attempt	2.26	1.79–3.97	0.815365	0.203202	Truancy / skipped school / unauthorised absence	Adjusted
Pengpid & Peltzer (2021), Tonga	Suicide attempt	2.72	1.66–4.75	1.000632	0.268196	Truancy / skipped school / unauthorised absence	Adjusted
Cheng et al. (2009)	Suicide attempt	2.89	1.51–5.26	1.061257	0.318373	Truancy / skipped school / unauthorised absence	Adjusted
Fergusson et al. (2003)	Suicide attempt	4.07	2.45–6.72	1.403643	0.257398	Truancy / skipped school / unauthorised absence	Adjusted

Note. The ORs, confidence intervals, log ORs, and standard errors in Supplementary Tables S3–S5 are the final values used in the analysis dataset. No values were manually transcribed into figures; Figures 3–5 were regenerated from the same dataset.
