# Supplementary material for: School Absenteeism and Self-Harm or Suicidal Outcomes Among Children, Adolescents, and School-Aged Young People: A Systematic Review and Meta-Analysis
Source: Children (Basel). 2026 Oct 10;13(10):1373. doi: 10.3390/children13101373 (PMC13703334; doi:10.3390/children13101373)
Supplement: Supplementary file 1 [file children-13-01373-s001.zip › Supplementary File S3. Risk-of-bias assessment table.pdf]

Supplementary Table S6. Study-level risk-of-bias assessment

Note. Ratings were extracted from the risk-of-bias spreadsheet. The source label “High” was harmonised to “Serious” to match ROBINS-style terminology used in the manuscript. “Deviations/timing-related bias” is used because school absenteeism was an observational exposure rather than an assigned intervention. The final column summarises the main domain-level reason for the overall judgement.

Study	Confounding	Selection bias	Exposure classification	Deviations/timing-related bias	Missing data	Outcome measurement	Selective reporting	Overall ROB	Main reason for overall judgement
Pandey et al., 2019, Nepal	Moderate	Moderate	Moderate	Low	Low	Moderate	Low	Moderate	Overall judgement reflects moderate concerns in confounding, selection bias, exposure classification, outcome measurement.
Pengpid & Peltzer, 2021, Trinidad	Moderate	Moderate	Moderate	Low	Moderate	Moderate	Low	Moderate	Overall judgement reflects moderate concerns in confounding, selection bias, exposure classification, missing data.
Pengpid & Peltzer, 2021, Tonga	Moderate	Low	Moderate	Low	Low	Moderate	Low	Moderate	Overall judgement reflects moderate concerns in confounding, exposure classification, outcome measurement.
Marthoenis et al., 2022, Bangladesh	Serious	Moderate	Moderate	Low	Serious	Serious	Moderate	Serious	Overall judgement driven by serious concerns in confounding, missing data, outcome measurement.
Sarfo et al., 2022, Saint Vincent	Moderate	Moderate	Moderate	Low	Low	Moderate	Low	Moderate	Overall judgement reflects moderate concerns in confounding, selection bias, exposure classification, outcome measurement.
Widnall et al., 2022, UK	Moderate	Moderate	Low	Low	Moderate	Moderate	Low	Moderate	Overall judgement reflects moderate concerns in confounding, selection bias, missing data, outcome measurement.
Diallo et al., 2023, Benin, Ghana	Moderate	Moderate	Moderate	Low	Moderate	Moderate	Low	Moderate	Overall judgement reflects moderate concerns in confounding, selection bias, exposure classification, missing data.
Forty et al., 2023, Botswana	Serious	Moderate	Moderate	Low	Moderate	Serious	Moderate	Serious	Overall judgement driven by serious concerns in confounding, outcome measurement.
Sarfo et al., 2023, Samoa	Moderate	Moderate	Moderate	Low	Moderate	Moderate	Low	Moderate	Overall judgement reflects moderate concerns in confounding, selection bias, exposure classification, missing data.
Dadras et al., 2024, Argentina	Serious	Serious	Moderate	Low	Moderate	Moderate	Low	Serious	Overall judgement driven by serious concerns in confounding, selection bias.
Sarfo et al., 2024, Benin	Moderate	Moderate	Moderate	Low	Low	Moderate	Low	Moderate	Overall judgement reflects moderate concerns in confounding, selection bias, exposure classification, outcome measurement.
Diogu et al., 2025, Wales, UK	Moderate	Low	Low	Low	Low	Low	Low	Moderate	Overall judgement reflects moderate concerns in confounding.
Sarfo et al., 2026, Bolivia	Moderate	Moderate	Moderate	Low	Low	Moderate	Low	Moderate	Overall judgement reflects moderate concerns in confounding, selection bias, exposure classification, outcome measurement.
Zeng et al., 2026, New Zealand	Moderate	Low	Low	Low	Low	Low	Low	Moderate	Overall judgement reflects moderate concerns in confounding.
Pham & Adesman, 2017, USA	Serious	Moderate	Moderate	Low	Moderate	Serious	Moderate	Serious	Overall judgement driven by serious concerns in confounding, outcome measurement.
Oppong Asante et al., 2017, Ghana	Moderate	Low	Moderate	Low	Low	Moderate	Low	Moderate	Overall judgement reflects moderate concerns in confounding, exposure classification, outcome measurement.
Bailey et al., 2015, USA	Serious	Moderate	Moderate	Low	Moderate	Low	Low	Serious	Overall judgement driven by serious concerns in confounding.
Bjarnason et al., 1994, Iceland	Serious	Moderate	Moderate	Low	Moderate	Moderate	Moderate	Serious	Overall judgement driven by serious concerns in confounding.
Borowsky et al., 1999, USA	Serious	Serious	Moderate	Low	Moderate	Moderate	Moderate	Serious	Overall judgement driven by serious concerns in confounding, selection bias.
Borowsky et al., 2001, USA	Moderate	Moderate	Moderate	Low	Moderate	Moderate	Moderate	Moderate	Overall judgement reflects moderate concerns in confounding, selection bias, exposure classification, missing data.
Brunner et al., 2014, 11 EU	Moderate	Serious	Moderate	Low	Moderate	Moderate	Low	Serious	Overall judgement driven by serious concerns in selection bias.
Chen et al., 2005, Malaysia	Serious	Moderate	Moderate	Low	Moderate	Moderate	Moderate	Serious	Overall judgement driven by serious concerns in confounding.
Cheng et al., 2009, China	Moderate	Moderate	Moderate	Low	Low	Moderate	Low	Moderate	Overall judgement reflects moderate concerns in confounding, selection bias, exposure classification, outcome measurement.
Lewinsohn et al., 1993, USA	Serious	Moderate	Moderate	Low	Moderate	Moderate	Moderate	Serious	Overall judgement driven by serious concerns in confounding.
Lewinsohn et al., 1994, USA	Moderate	Moderate	Moderate	Low	Moderate	Moderate	Moderate	Moderate	Overall judgement reflects moderate concerns in confounding, selection bias, exposure classification, missing data.
Lyon et al., 2000, USA	Serious	Serious	Serious	Low	Moderate	Low	Moderate	Serious	Overall judgement driven by serious concerns in confounding, selection bias, exposure classification.
Noble et al., 2011, USA	Serious	Moderate	Moderate	Low	Low	Serious	Moderate	Serious	Overall judgement driven by serious concerns in confounding, outcome measurement.
Pagès et al., 2004, France	Serious	Moderate	Moderate	Low	Low	Moderate	Moderate	Serious	Overall judgement driven by serious concerns in confounding.
Pillai et al., 2009, India	Serious	Serious	Serious	Low	Moderate	Moderate	Low	Serious	Overall judgement driven by serious concerns in confounding, selection bias, exposure classification.
Randall et al., 2014, Benin	Moderate	Low	Moderate	Low	Moderate	Moderate	Low	Moderate	Overall judgement reflects moderate concerns in confounding, exposure classification, missing data, outcome measurement.
Sharma et al., 2015, Peru	Moderate	Moderate	Moderate	Low	Moderate	Moderate	Moderate	Moderate	Overall judgement reflects moderate concerns in confounding, selection bias, exposure classification, missing data.
Taliaferro et al., 2014, USA	Serious	Moderate	Serious	Low	Moderate	Moderate	Moderate	Serious	Overall judgement driven by serious concerns in confounding, exposure classification.
Wilson et al., 2012, Seychelles	Moderate	Moderate	Moderate	Low	Low	Moderate	Low	Moderate	Overall judgement reflects moderate concerns in confounding, selection bias, exposure classification, outcome measurement.
Xin et al., 2017, China	Moderate	Moderate	Moderate	Low	Low	Moderate	Moderate	Moderate	Overall judgement reflects moderate concerns in confounding, selection bias, exposure classification, outcome measurement.
Fergusson et al., 2003, New Zealand	Moderate	Moderate	Moderate	Low	Moderate	Low	Moderate	Moderate	Overall judgement reflects moderate concerns in confounding, selection bias, exposure classification, missing data.
Peltzer & Pengpid, 2017	Moderate	Moderate	Moderate	Low	Moderate	Moderate	Low	Moderate	Overall judgement reflects moderate concerns in confounding, selection bias, exposure classification, missing data.
Almansour & Siziya, 2017, Swaziland	Moderate	Low	Moderate	Low	Low	Moderate	Low	Moderate	Overall judgement reflects moderate concerns in confounding, exposure classification, outcome measurement.
Choquet & Menke, 1990, France	Serious	Moderate	Moderate	Low	Moderate	Moderate	Moderate	Serious	Overall judgement driven by serious concerns in confounding.
Cwik et al., 2015	Serious	Serious	Serious	Low	Moderate	Low	Moderate	Serious	Overall judgement driven by serious concerns in confounding, selection bias, exposure classification.
Davaasambuu et al., 2017, Mongolia	Moderate	Low	Moderate	Low	Low	Moderate	Low	Moderate	Overall judgement reflects moderate concerns in confounding, exposure classification, outcome measurement.
de Man et al., 1993, Canada	Moderate	Low	Low	Low	Low	Low	Moderate	Moderate	Overall judgement reflects moderate concerns in confounding, selective reporting.
Donath et al., 2014, Germany	Moderate	Moderate	Moderate	Low	Moderate	Moderate	Low	Moderate	Overall judgement reflects moderate concerns in confounding, selection bias, exposure classification, missing data.
Epstein & Spirito, 2009, USA	Serious	Moderate	Serious	Low	Moderate	Moderate	Moderate	Serious	Overall judgement driven by serious concerns in confounding, exposure classification.
Evren et al., 2014, Turkey	Serious	Moderate	Moderate	Low	Moderate	Moderate	Moderate	Serious	Overall judgement driven by serious concerns in confounding.
Kandel et al., 1991, USA	Serious	Serious	Serious	Low	Moderate	Moderate	Moderate	Serious	Overall judgement driven by serious concerns in confounding, selection bias, exposure classification.

Study	Confounding	Selection bias	Exposure classification	Deviations/timing-related bias	Missing data	Outcome measurement	Selective reporting	Overall ROB	Main reason for overall judgement
Larsson & Sund, 2008, Norway	Moderate	Moderate	Moderate	Low	Moderate	Moderate	Low	Moderate	Overall judgement reflects moderate concerns in confounding, selection bias, exposure classification, missing data.
Lau et al., 2010, China	Moderate	Serious	Moderate	Low	Low	Moderate	Moderate	Serious	Overall judgement driven by serious concerns in selection bias.

Summary: 47 studies were assessed; 26 were rated as moderate overall risk of bias and 21 as serious overall risk of bias. No study was rated as low overall risk of bias.
