# Supplementary material for: School Absenteeism and Self-Harm or Suicidal Outcomes Among Children, Adolescents, and School-Aged Young People: A Systematic Review and Meta-Analysis
Source: Children (Basel). 2026 Oct 10;13(10):1373. doi: 10.3390/children13101373 (PMC13703334; doi:10.3390/children13101373)
Supplement: Supplementary file 1 [file children-13-01373-s001.zip › Supplementary File S4. Dataset-overlap assessment, sensitivity analyses, and certainty-of-evidence judgements.pdf]

Supplementary File S4. Dataset-overlap assessment, sensitivity analyses, and certainty-of-evidence judgements

This file provides the dataset-overlap assessment, numerical sensitivity analyses, subgroup-analysis feasibility statements, and domain-level certainty-of-evidence judgements. All numerical meta-analytic estimates in this file were generated from the final analytical dataset reported in Supplementary Tables S3–S5 and Supplementary File S6.

Supplementary Table S7. Study-level dataset overlap assessment

Study	Country / dataset details	Potential dataset overlap assessment	Decision for quantitative synthesis
Almansour & Siziya, 2017 (1)	Swaziland; 2013; Cross-sectional secondary analysis	No apparent overlap identified from extracted country, dataset year/wave, and study design information.	Included in at least one primary pooled meta-analysis as an extracted estimate.
Oppong Asante et al., 2017 (2)	Ghana; 2012; Cross-sectional secondary analysis	Potential overlap with Diallo et al. (2023), because Diallo et al. included a Ghana GSHS component that may overlap with the Ghana 2012 GSHS dataset.	Included in primary analysis; retained as preferred country-specific Ghana estimate. Dependency assessed by sensitivity analysis excluding Diallo et al. (2023).
Bailey et al., 2015(3)	USA; 2006-2010; Retrospective record-linkage cohort / mortality study	No apparent overlap identified from extracted country, dataset year/wave, and study design information.	Not included in pooled meta-analysis; retained for qualitative/narrative synthesis.
Bjarnason et al., 1994(4)	Iceland; 1992; Cross-sectional population-based school survey	No apparent overlap identified from extracted country, dataset year/wave, and study design information.	Not included in pooled meta-analysis; retained for qualitative/narrative synthesis.
Borowsky et al., 1999(5)	USA; 1990; Cross-sectional school survey	No apparent overlap identified from extracted country, dataset year/wave, and study design information.	Not included in pooled meta-analysis; retained for qualitative/narrative synthesis.
Borowsky et al., 2001(6)	USA; 1995-1996; Longitudinal cohort	No apparent overlap identified from extracted country, dataset year/wave, and study design information.	Not included in pooled meta-analysis; retained for qualitative/narrative synthesis.
Brunner et al., 2014(7)	EU; NR; Cross-sectional multicountry school survey	No apparent overlap identified from extracted country, dataset year/wave, and study design information.	Not included in pooled meta-analysis; retained for qualitative/narrative synthesis.
Chen et al., 2005(8)	Malaysia; 2001; Cross-sectional school survey	No apparent overlap identified from extracted country, dataset year/wave, and study design information.	Not included in pooled meta-analysis; retained for qualitative/narrative synthesis.
Cheng et al., 2009 (9)	China; 2003; Cross-sectional school survey	No apparent overlap identified from extracted country, dataset year/wave, and study design information.	Included in at least one primary pooled meta-analysis as an extracted estimate.
Choquet & Menke, 1990 (10)	France; 1988; Cross-sectional school survey	No apparent overlap identified from extracted country, dataset year/wave, and study design information.	Not included in pooled meta-analysis; retained for qualitative/narrative synthesis.
Cwik et al., 2015 (11)	USA; NR; Cross-sectional community sample of adolescent suicide attempters	No apparent overlap identified from extracted country, dataset year/wave, and study design information.	Not included in pooled meta-analysis; retained for qualitative/narrative synthesis.
Davaasambuu et al., 2017 (12)	Mongolia; 2013; Cross-sectional	No apparent overlap identified from extracted country, dataset year/wave,	Included in at least one primary pooled meta-analysis as an extracted

	school survey	and study design information.	estimate.
de Man et al., 1993(13)	Canada; NR; Cross-sectional school survey	No apparent overlap identified from extracted country, dataset year/wave, and study design information.	Not included in pooled meta-analysis; retained for qualitative/narrative synthesis.
Donath et al., 2014 (14)	Germany; 2007-2008; Cross-sectional representative school survey	No apparent overlap identified from extracted country, dataset year/wave, and study design information.	Included in at least one primary pooled meta-analysis as an extracted estimate.
Epstein & Spirito, 2009 (15)	USA; 2005; Cross-sectional secondary analysis	No apparent overlap identified from extracted country, dataset year/wave, and study design information.	Not included in pooled meta-analysis; retained for qualitative/narrative synthesis.
Evren et al., 2014 (16)	Turkey; 2012; Cross-sectional online school survey	No apparent overlap identified from extracted country, dataset year/wave, and study design information.	Not included in pooled meta-analysis; retained for qualitative/narrative synthesis.
Kandel et al., 1991 (17)	USA; 1986; Cross-sectional school survey	No apparent overlap identified from extracted country, dataset year/wave, and study design information.	Not included in pooled meta-analysis; retained for qualitative/narrative synthesis.
Larsson & Sund, 2008 (18)	Norway; 1998-1999; Prospective cohort school survey	No apparent overlap identified from extracted country, dataset year/wave, and study design information.	Not included in pooled meta-analysis; retained for qualitative/narrative synthesis.
Lau et al., 2010 (19)	China; 2008; Cross-sectional school survey	No apparent overlap identified from extracted country, dataset year/wave, and study design information.	Not included in pooled meta-analysis; retained for qualitative/narrative synthesis.
Lewinsohn et al., 1993(20)	USA; 1987-1989; Cross-sectional baseline analysis within community cohort	No apparent overlap identified from extracted country, dataset year/wave, and study design information.	Not included in pooled meta-analysis; retained for qualitative/narrative synthesis.
Lewinsohn et al., 1994(21)	USA; 1987-1990; Prospective cohort	No apparent overlap identified from extracted country, dataset year/wave, and study design information.	Not included in pooled meta-analysis; retained for qualitative/narrative synthesis.
Lyon et al., 2000 (22)	USA; NR; Matched case-control clinical study	No apparent overlap identified from extracted country, dataset year/wave, and study design information.	Not included in pooled meta-analysis; retained for qualitative/narrative synthesis.
Noble et al., 2011(23)	USA; NR; Matched case-control within cross-sectional school survey	No apparent overlap identified from extracted country, dataset year/wave, and study design information.	Not included in pooled meta-analysis; retained for qualitative/narrative synthesis.
Pages et al., 2004 (24)	France; 1999; Cross-sectional national school survey	No apparent overlap identified from extracted country, dataset year/wave, and study design information.	Not included in pooled meta-analysis; retained for qualitative/narrative synthesis.
Pillai et al., 2009 (25)	India; 2006; Cross-sectional community survey	No apparent overlap identified from extracted country, dataset year/wave, and study design information.	Not included in pooled meta-analysis; retained for qualitative/narrative synthesis.
Randall et al., 2014 (26)	Benin; 2009; Cross-sectional school survey; multinomial logistic regression	Benin GSHS, but no apparent overlap with Sarfo et al. (2024) or Diallo et al. (2023) because the extracted survey year differs.	Not included in pooled meta-analysis; retained for qualitative/narrative synthesis.
Sharma et al., 2015 (27)	Peru; 2014; Cross-sectional school survey	No apparent overlap identified from extracted country, dataset year/wave, and study design information.	Not included in pooled meta-analysis; retained for qualitative/narrative synthesis.
Taliaferro et al., 2014(28)	USA; 2010; Cross-sectional population-based school survey	No apparent overlap identified from extracted country, dataset year/wave, and study design information.	Not included in pooled meta-analysis; retained for qualitative/narrative synthesis.

Wilson et al., 2012 (29)	Seychelles; NR; Cross-sectional GSHS secondary analysis	No apparent overlap identified from extracted country, dataset year/wave, and study design information.	Not included in pooled meta-analysis; retained for qualitative/narrative synthesis.
Xin et al., 2017 (30)	China; NR; Cross-sectional school-based survey	No apparent overlap identified from extracted country, dataset year/wave, and study design information.	Not included in pooled meta-analysis; retained for qualitative/narrative synthesis.
Fergusson et al., 2003 (31)	New Zealand; 1977-1998; Prospective longitudinal birth cohort	No apparent overlap identified from extracted country, dataset year/wave, and study design information.	Included in at least one primary pooled meta-analysis as an extracted estimate.
Peltzer & Pengpid, 2017(32)	Cambodia; 2007-2013; Cross-sectional pooled GSHS secondary analysis	Pooled/multicountry GSHS-type analysis; no overlapping country-specific estimate from the same country-year was included in the same pooled outcome synthesis.	Included in at least one primary pooled meta-analysis as an extracted estimate.
Pandey et al., 2019 (33)	Nepal; 2015; Cross-sectional secondary analysis	No apparent overlap identified from extracted country, dataset year/wave, and study design information.	Included in at least one primary pooled meta-analysis as an extracted estimate.
Pengpid & Peltzer, 2021(34)	Trinidad; 2017; Cross-sectional secondary analysis	No apparent overlap identified from extracted country, dataset year/wave, and study design information.	Included in at least one primary pooled meta-analysis as an extracted estimate.
Pengpid & Peltzer, 2021(35)	Tonga; 2017; Cross-sectional secondary analysis	No apparent overlap identified from extracted country, dataset year/wave, and study design information.	Included in at least one primary pooled meta-analysis as an extracted estimate.
Marthoenis et al., 2022(36)	Bangladesh; 2014-2015; Cross-sectional secondary analysis	No apparent overlap identified from extracted country, dataset year/wave, and study design information.	Not included in pooled meta-analysis; retained for qualitative/narrative synthesis.
Sarfo et al., 2022(37)	Saint Vincent; 2018; Cross-sectional secondary analysis	No apparent overlap identified from extracted country, dataset year/wave, and study design information.	Included in at least one primary pooled meta-analysis as an extracted estimate.
Widnall et al., 2022(38)	UK; 2009-2013; Retrospective cohort / linked administrative data study	Potential overlap with Diogu et al. (2025) because both studies used large-scale UK/Wales linked administrative records and had partially overlapping calendar periods. The extent to which the same data infrastructure or population contributed to both studies could not be fully confirmed from the published reports.	Not included in pooled meta-analysis; retained for qualitative/narrative synthesis. Potential dependency with Diogu et al. (2025) was flagged in the dataset-overlap assessment and interpreted cautiously.
Diallo et al., 2023 (39)	Benin; 2012-2017; Cross-sectional pooled secondary analysis	Potential overlap with country-specific Ghana and Benin GSHS estimates reported by Oppong Asante et al. (2017) and Sarfo et al. (2024).	Included in primary analysis; excluded in independent-estimate sensitivity analyses for suicidal ideation and suicide attempt.
Forty et al., 2023(40)	Botswana; 2010; Cross-sectional secondary analysis	No apparent overlap identified from extracted country, dataset year/wave, and study design information.	Included in at least one primary pooled meta-analysis as an extracted estimate.
Sarfo et al., 2023(41)	Samoa; 2017; Cross-sectional secondary analysis	No apparent overlap identified from extracted country, dataset year/wave, and study design information.	Included in at least one primary pooled meta-analysis as an extracted estimate.
Dadras et al., 2024(42)	Argentina; 2018; Cross-sectional secondary analysis; restricted to adolescents with suicidal thoughts	No apparent overlap identified from extracted country, dataset year/wave, and study design information.	Not included in pooled meta-analysis; retained for qualitative/narrative synthesis.

Sarfo et al., 2024(43)	Benin; 2016; Cross-sectional secondary analysis	Potential overlap with Diallo et al. (2023), because both appear to include a Benin GSHS component from the same survey period.	Included in primary analysis; retained as preferred country-specific Benin estimate. Dependency assessed by sensitivity analysis excluding Diallo et al. (2023).
Diogu et al., 2025(44)	Wales, UK; 2012/13-2021; Retrospective electronic cohort / record-linkage study	Potential overlap with Widnall et al. (2022) because both studies used large-scale UK/Wales linked administrative records and had partially overlapping calendar periods. The extent to which the same data infrastructure or population contributed to both studies could not be fully confirmed from the published reports.	Not included in pooled meta-analysis; retained for qualitative/narrative synthesis. Potential dependency with Widnall et al. (2022) was flagged in the dataset-overlap assessment and interpreted cautiously.
Sarfo et al., 2026(45)	Bolivia; 2018; Cross-sectional secondary analysis	No apparent overlap identified from extracted country, dataset year/wave, and study design information.	Included in at least one primary pooled meta-analysis as an extracted estimate.
Zeng et al., 2026(46)	New Zealand; 2015-2021; Retrospective longitudinal matched case-control / nested case-control study	No apparent overlap identified from extracted country, dataset year/wave, and study design information.	Not included in pooled meta-analysis; retained for qualitative/narrative synthesis.
Pham & Adesman, 2017 (47)	USA; 2015; Cross-sectional secondary analysis	No apparent overlap identified from extracted country, dataset year/wave, and study design information.	Not included in pooled meta-analysis; retained for qualitative/narrative synthesis.

Note. The overlap assessment was based on extracted country, dataset/survey system, survey year or wave, sampling frame, exposure, outcome, and authorship information. When potential overlap was identified, it was evaluated in an independent-estimate sensitivity analysis.

Supplementary Table S8. Numerical sensitivity analyses and subgroup-analysis feasibility

Outcome	Analysis / subgroup variable	k	Pooled OR	95% CI / prediction interval	I²	Interpretation / reason for non-analysis
Suicidal ideation	Model sensitivity: common-effect model	12	1.26	1.22–1.31	63.3%	Model-sensitivity estimate generated from the final analytical dataset.
Suicidal ideation	Primary model: DerSimonian–Laird random-effects model	12	1.32	1.19–1.46	63.3%	Primary random-effects estimate reported in the manuscript.
Suicidal ideation	Model sensitivity: REML random-effects model	12	1.33	1.17–1.51	63.3%	Alternative tau-squared estimator.
Suicidal ideation	Model sensitivity: REML with Hartung–Knapp-type confidence interval	12	1.33	1.16–1.53	63.3%	Hartung–Knapp-type confidence interval used as a robustness check.
Suicidal ideation	Prediction interval based on REML random-effects model	12	1.33	0.89–2.00	63.3%	Prediction interval indicates the expected range of effects in a new population.
Suicidal ideation	Overlap sensitivity: excluding Diallo et al. (2023)	11	1.29	1.17–1.43	62.7%	Addresses potential overlap between the pooled West African GSHS analysis and country-specific

						Ghana/Benin estimates.
Suicide plan	Model sensitivity: common-effect model	6	1.31	1.21–1.42	0.0%	Model-sensitivity estimate generated from the final analytical dataset.
Suicide plan	Primary model: DerSimonian–Laird random-effects model	6	1.31	1.21–1.42	0.0%	Primary random-effects estimate reported in the manuscript.
Suicide plan	Model sensitivity: REML random-effects model	6	1.31	1.21–1.42	0.0%	Alternative tau-squared estimator.
Suicide plan	Model sensitivity: REML with Hartung–Knapp-type confidence interval	6	1.31	1.19–1.45	0.0%	Hartung–Knapp-type confidence interval used as a robustness check.
Suicide plan	Prediction interval based on REML random-effects model	6	1.31	1.18–1.46	0.0%	Prediction interval indicates the expected range of effects in a new population.
Suicide plan	Overlap sensitivity: excluding potentially overlapping GSHS pooled-country estimates	0 excluded; k = 6	Not required	Not applicable	0.0%	No potentially overlapping pooled-country estimate was identified for this synthesis.
Suicide attempt	Model sensitivity: common-effect model	14	1.52	1.44–1.60	69.4%	Model-sensitivity estimate generated from the final analytical dataset.
Suicide attempt	Primary model: DerSimonian–Laird random-effects model	14	1.60	1.41–1.81	69.4%	Primary random-effects estimate reported in the manuscript.
Suicide attempt	Model sensitivity: REML random-effects model	14	1.64	1.40–1.93	69.4%	Alternative tau-squared estimator.
Suicide attempt	Model sensitivity: REML with Hartung–Knapp-type confidence interval	14	1.64	1.36–1.99	69.4%	Hartung–Knapp-type confidence interval used as a robustness check.
Suicide attempt	Prediction interval based on REML random-effects model	14	1.64	0.92–2.92	69.4%	Prediction interval indicates the expected range of effects in a new population.
Suicide attempt	Overlap sensitivity: excluding Diallo et al. (2023)	13	1.63	1.42–1.86	71.4%	Addresses potential overlap between the pooled West African GSHS analysis and country-specific Ghana/Benin estimates.
Suicidal ideation	Subgroup: exposure definition — truancy/skipped school/unauthorised	12	1.32	1.19–1.46	63.3%	Numerical subgroup result from the final analytical dataset where at least three

	absence					estimates were available.
Suicidal ideation	Subgroup: exposure definition — other attendance-related measure	0	Not pooled	Not applicable	Not applicable	Not pooled because fewer than three comparable estimates were available.
Suicidal ideation	Subgroup: exposure definition — chronic/persistent absence	0	Not pooled	Not applicable	Not applicable	No sufficiently comparable estimates for this outcome were available in the final quantitative dataset.
Suicidal ideation	Subgroup: exposure definition — school refusal/avoidance	0	Not pooled	Not applicable	Not applicable	No sufficiently comparable estimates for this outcome were available in the final quantitative dataset.
Suicidal ideation	Subgroup: exposure definition — absence because of feeling unsafe	0	Not pooled	Not applicable	Not applicable	No sufficiently comparable estimates for this outcome were available in the final quantitative dataset.
Suicide plan	Subgroup: exposure definition — truancy/skipped school/unauthorised absence	6	1.31	1.21–1.42	0.0%	Numerical subgroup result from the final analytical dataset where at least three estimates were available.
Suicide plan	Subgroup: exposure definition — other attendance-related measure	0	Not pooled	Not applicable	Not applicable	Not pooled because fewer than three comparable estimates were available.
Suicide plan	Subgroup: exposure definition — chronic/persistent absence	0	Not pooled	Not applicable	Not applicable	No sufficiently comparable estimates for this outcome were available in the final quantitative dataset.
Suicide plan	Subgroup: exposure definition — school refusal/avoidance	0	Not pooled	Not applicable	Not applicable	No sufficiently comparable estimates for this outcome were available in the final quantitative dataset.
Suicide plan	Subgroup: exposure definition — absence because of feeling unsafe	0	Not pooled	Not applicable	Not applicable	No sufficiently comparable estimates for this outcome were available in the final quantitative dataset.
Suicide attempt	Subgroup: exposure definition — truancy/skipped school/unauthorised absence	13	1.64	1.40–1.92	71.0%	Numerical subgroup result from the final analytical dataset where at least three estimates were available.
Suicide attempt	Subgroup: exposure definition — other attendance-related	1	Not pooled	Not applicable	Not applicable	Not pooled because fewer than three comparable estimates

	measure					were available.
Suicide attempt	Subgroup: exposure definition — chronic/persistent absence	0	Not pooled	Not applicable	Not applicable	No sufficiently comparable estimates for this outcome were available in the final quantitative dataset.
Suicide attempt	Subgroup: exposure definition — school refusal/avoidance	0	Not pooled	Not applicable	Not applicable	No sufficiently comparable estimates for this outcome were available in the final quantitative dataset.
Suicide attempt	Subgroup: exposure definition — absence because of feeling unsafe	0	Not pooled	Not applicable	Not applicable	No sufficiently comparable estimates for this outcome were available in the final quantitative dataset.
Suicidal ideation	Subgroup: study design — cross-sectional studies	11	1.25	1.16–1.35	36.2%	Numerical subgroup result where at least three estimates were available.
Suicidal ideation	Subgroup: study design — longitudinal/cohort/record-linkage studies	1	Not pooled	Not applicable	Not applicable	Not pooled where fewer than three sufficiently comparable estimates were available.
Suicide plan	Subgroup: study design — cross-sectional studies	6	1.31	1.21–1.42	0.0%	Numerical subgroup result where at least three estimates were available.
Suicide plan	Subgroup: study design — longitudinal/cohort/record-linkage studies	0	Not pooled	Not applicable	Not applicable	Not pooled where fewer than three sufficiently comparable estimates were available.
Suicide attempt	Subgroup: study design — cross-sectional studies	13	1.51	1.36–1.68	56.4%	Numerical subgroup result where at least three estimates were available.
Suicide attempt	Subgroup: study design — longitudinal/cohort/record-linkage studies	1	Not pooled	Not applicable	Not applicable	Not pooled where fewer than three sufficiently comparable estimates were available.
Suicidal ideation	Subgroup: data source	Not separately pooled	Not pooled	Not applicable	Not applicable	Most pooled estimates used self-reported survey or cohort measures; administrative/clinical strata contained too few comparable estimates for outcome-specific pooling.
Suicidal ideation	Subgroup: adjustment level	Not separately pooled	Not pooled	Not applicable	Not applicable	All estimates retained in the final quantitative dataset

						were classified as adjusted; no unadjusted/minimally adjusted comparator stratum was available.
Suicidal ideation	Subgroup: age group	Not separately pooled	Not pooled	Not applicable	Not applicable	Study-level estimates were not consistently reported by age category, and sex/age-stratified ORs were not sufficiently available for outcome-specific pooling.
Suicidal ideation	Subgroup: sex/gender	Not separately pooled	Not pooled	Not applicable	Not applicable	Sex/gender-stratified attendance-specific estimates were not consistently available; this subgroup analysis was therefore not conducted.
Suicidal ideation	Subgroup: geographical region	Not separately pooled	Not pooled	Not applicable	Not applicable	Country and regional coverage was heterogeneous, but prespecified region strata had sparse and non-comparable estimates within outcome categories; no region-specific pooled subgroup analysis was conducted.
Suicide plan	Subgroup: data source	Not separately pooled	Not pooled	Not applicable	Not applicable	Most pooled estimates used self-reported survey or cohort measures; administrative/clinical strata contained too few comparable estimates for outcome-specific pooling.
Suicide plan	Subgroup: adjustment level	Not separately pooled	Not pooled	Not applicable	Not applicable	All estimates retained in the final quantitative dataset were classified as adjusted; no unadjusted/minimally adjusted comparator stratum was available.
Suicide plan	Subgroup: age group	Not separately pooled	Not pooled	Not applicable	Not applicable	Study-level estimates were not consistently reported by age category, and sex/age-stratified ORs were not sufficiently available for outcome-specific pooling.

Suicide plan	Subgroup: sex/gender	Not separately pooled	Not pooled	Not applicable	Not applicable	Sex/gender-stratified attendance-specific estimates were not consistently available; this subgroup analysis was therefore not conducted.
Suicide plan	Subgroup: geographical region	Not separately pooled	Not pooled	Not applicable	Not applicable	Country and regional coverage was heterogeneous, but prespecified region strata had sparse and non-comparable estimates within outcome categories; no region-specific pooled subgroup analysis was conducted.
Suicide attempt	Subgroup: data source	Not separately pooled	Not pooled	Not applicable	Not applicable	Most pooled estimates used self-reported survey or cohort measures; administrative/clinical strata contained too few comparable estimates for outcome-specific pooling.
Suicide attempt	Subgroup: adjustment level	Not separately pooled	Not pooled	Not applicable	Not applicable	All estimates retained in the final quantitative dataset were classified as adjusted; no unadjusted/minimally adjusted comparator stratum was available.
Suicide attempt	Subgroup: age group	Not separately pooled	Not pooled	Not applicable	Not applicable	Study-level estimates were not consistently reported by age category, and sex/age-stratified ORs were not sufficiently available for outcome-specific pooling.
Suicide attempt	Subgroup: sex/gender	Not separately pooled	Not pooled	Not applicable	Not applicable	Sex/gender-stratified attendance-specific estimates were not consistently available; this subgroup analysis was therefore not conducted.
Suicide attempt	Subgroup: geographical region	Not separately pooled	Not pooled	Not applicable	Not applicable	Country and regional coverage was heterogeneous, but prespecified region strata had sparse and non-comparable estimates within outcome categories;

							no region-specific pooled subgroup analysis was conducted.

Note. Numerical values in Supplementary Table S8 were calculated from the same final analytical dataset used for Supplementary Tables S3–S5 and Figures 3–5. Subgroup analyses were not pooled when fewer than three sufficiently comparable estimates were available or when study-level/stratified estimates were not reported in a form suitable for pooling.

Supplementary Table S10. Certainty-of-evidence domain judgements by pooled outcome

Outcome	Risk of bias	Inconsistency	Indirectness	Imprecision	Reporting/publication bias	Overall certainty	Rationale
Suicidal ideation	Serious concerns: no included study was judged low overall risk of bias; residual confounding and cross-sectional timing were common.	Serious concerns: moderate-to-substantial heterogeneity (I^2 = 63.3%) and variation in effect magnitude.	Some concerns: population and outcome generally aligned, but exposure was predominantly truancy/skipped/unauthorised absence rather than all attendance constructs.	Not serious: confidence interval around primary pooled estimate excluded the null, but prediction interval crossed the null.	Some concerns: funnel plot interpretation limited by small number of estimates and selective availability of attendance-specific estimates.	Low	Low-certainty evidence suggests an association, but causal inference is limited.
Suicide plan	Serious concerns: no included study was judged low overall risk of bias; residual confounding remained possible.	Not serious: no meaningful heterogeneity in the primary analysis (I^2 = 0.0%).	Some concerns: estimates predominantly represented truancy/skipped/unauthorised absence.	Not serious: confidence interval and prediction interval excluded the null.	Some concerns: funnel plot interpretation limited because only six estimates were available.	Low	Low-certainty evidence suggests an association; certainty was not upgraded because evidence was observational and bias concerns remained.
Suicide attempt	Serious concerns: no included study was judged low overall risk of bias; residual confounding and cross-sectional timing were common.	Serious concerns: moderate-to-substantial heterogeneity (I^2 = 69.4%) and wide prediction interval.	Some concerns: most estimates represented truancy/skipped/unauthorised absence, with limited evidence for broader attendance constructs.	Some concerns: confidence interval excluded the null, but prediction interval crossed the null.	Some concerns: funnel plot interpretation limited by heterogeneity and potential selective availability of attendance-specific estimates.	Low	Low-certainty evidence suggests an association; heterogeneity and risk of bias limit certainty.

Note. Certainty judgements were made using an adapted GRADE approach for observational evidence and correspond to the procedure described in Methods Section 2.9 of the manuscript.
